# The Cerebellar Role in Emotions at a Turning Point: Bibliometric Analysis and Collaboration Networks

**DOI:** 10.1007/s12311-025-01800-7

**Published:** 2025-02-17

**Authors:** Dianela A. Osorio-Becerra, Egidio D’Angelo, Claudia Casellato

**Affiliations:** 1 Department of Brain and Behavioral Sciences, University of Pavia, Pavia, Italy; 2 Digital Neuroscience Center, IRCCS Mondino Foundation, Pavia, Italy

**Keywords:** Cerebellum, Emotions, Bibliometric analysis, Collaboration networks

## Abstract

The neural basis of emotional experience, both in neurotypical and clinical conditions, remains an open research topic. Historically, the cerebellum was considered a purely motor structure; however, studies since the mid-twentieth century and contributions like the cerebellar cognitive-affective syndrome, evidenced its role in emotion. This has led to an expansion of the paradigm, encouraging further research into the cerebellar role in emotion. Understanding this field's development is essential to assessing its current state, identifying knowledge gaps, and exploring emerging areas. This paper analyzes the evolution of scientific production, addressing how scientific interest has changed over time, factors driving growth, dominant topics, leading figures, and collaboration networks. This analysis identifies trends and opportunities, guiding strategies and advancing knowledge through a comprehensive view of the state-of-the-art in this research area. To achieve this, a systematic search was conducted in key databases, identifying 1,162 publications with which an exhaustive quantitative analysis was conducted using bibliometric techniques, network analysis, and visualization tools. The results show exponential growth in the field, evidenced by the increase in publications, researchers, funding sources, and the emergence of new topics. This interest, along with an interdisciplinary approach, has fostered collaboration, with large teams and multicenter projects emerging, although small, isolated teams still predominate. Research mainly focuses on neurological and affective disorders, with a predominance of studies in humans, followed by rodent models. Overall, the analysis reveals a highly interdisciplinary and expanding field. However, challenges remain, including unequal access to resources and limited exploration of some topics.

**Supplementary Information:**

The online version contains supplementary material available at 10.1007/s12311-025-01800-7.

## Introduction

The cerebellar role in emotion has emerged as a critical area of research in recent decades. Indeed, in parallel with the recognition of its central role in motor learning and control [1], numerous clinical, experimental, neuroimaging, and neurophysiological studies have shown that the cerebellum plays a crucial role in cognition and emotional processing [2–5]. In this context, its regulatory function in emotions such as anxiety and stress, its role in social perception through the interpretation of facial expressions and emotional tones, as well as its contribution to emotional memory and motivation, and its involvement in various psychiatric disorders [6–11], for example, have been demonstrated.

The growing interest in this field motivates this study, whose purpose is to critically evaluate the current state of knowledge, identify gaps and emerging trends, and guide future strategies. We propose a method that combines bibliometric analysis with collaboration network analysis, following a systematic search based on a clear and detailed methodological framework. This approach ensures a comprehensive study while promoting the reproducibility of results, thereby advancing research rigor and transparency.

Bibliometric analysis [12] and collaboration network analysis [13] are key tools that offer a clear view of the field's trends and dynamics.

Bibliometrics uses mathematical and statistical methods to analyze the scientific literature, identifying trends and characteristics of a specific discipline. A comprehensive view of the research landscape is obtained by considering elements such as publications, topics, authors, citations, and affiliations.

Meanwhile, integrated through complex network analysis and graph theory, collaboration networks offer an in-depth understanding of scientific collaborations, highlighting key patterns and dynamics in these interactions.

We employ a systematic approach to enhance the clarity, organization, and rigor of our research by combining a search framework (3WH) with the PRISMA statement. The 3WH framework helps define research questions, organize the search process, and focus on relevant aspects, ensuring comprehensive data collection. PRISMA complements this by providing clear guidelines for reporting the review process, including inclusion and exclusion criteria.

Our purpose with this analysis is not only to trace the evolution of knowledge on the cerebellar role in emotions but also to serve as a reference resource for people interested in exploring the field and its existing connections.

## Materials and Methods

### Search Frame

None of the most common search frameworks [14] are fully suitable for our purposes, therefore we have adapted the existing framework 3WH to better structure and direct our research questions, by adding the Why “factor” (Table 1).

**Table 1:** 3WH* framework applied to the research question

What	Why	When	Who	How
**What is the current state of research on the cerebellum's role in emotional processing?** -This question is open-ended and allows for a broad exploration of the topic -Focuses the research specifically on the cerebellum's role in emotions	**Why does this research matter?** -Address a gap in understanding -Synthesize existing knowledge, creating a much-needed overview of this emerging field -Provide a foundation for future research -Highlight clinical potential and therapeutic implications	**Timeline** -From the first available publication to the present	**Who are the research subjects and studies to be considered?** -Studies involving any species and approaches (experimental, clinical, computational…), discipline, or field of study	**How to conduct the investigation?** -Identify the corpus of literature over time by conducting a systematic search -Identify how the focus of research has changed over the years, map out existing publications, reveal trends, influential authors, and potential research areas -Gain a deeper understanding of the social dynamics within the field, revealing the collaborative landscape -Graphic visualization

* 3WH: What (topical), Who (population), When(temporal), and How (methodological); Why (purpose) has been added

### Database Identification

In our study, 4 highly known bibliographic reference databases have been considered, the most commonly used search engine in biomedicine: PubMed [15] and ScienceDirect [16], and the multidisciplinary databases Scopus [17] and Web of Science [18].

In all databases, the same criteria were employed, using Boolean operators and keywords: emotion, emotions, emotional, feeling, feelings, affect, affective, cerebellum, cerebellar. Specifically, the search builder in each database was:

- In PubMed (“emotions”[All Fields] OR “emotions”[MeSH Terms] OR “emotions”[All Fields] OR “emotion”[All Fields] OR “emotional”[All Fields] OR “feeling”[All Fields] OR “feelings”[All Fields] OR “affect”[All Fields] OR “affective”[All Fields]) AND (“cerebellum”[MeSH Terms] OR “cerebellum”[All Fields] OR “cerebellums”[All Fields] OR “cerebellum s”[All Fields] OR “cerebellar”[All Fields]).
- In Scopus, TITLE-ABS-KEY(emotion OR emotions OR emotional OR feeling OR feelings OR affect OR affective) AND TITLE-ABS-KEY(cerebellum OR cerebellums OR cerebellar).
- In Web of Science (ALL = (emotions OR emotion OR emotional OR feeling feelings OR affect OR affective) AND ALL = (cerebellum OR cerebellums OR cerebellar)).
- In ScienceDirect (emotion OR emotional OR feeling OR affect OR affective) AND (cerebellum OR cerebellar).

As the complete evolution of this area is of interest, no restriction in the search or downloading of data in terms of time limit, language limit, or type of document (journals, case studies, meta-analyzes, reviews, books, conference papers, etc.) were imposed.

At the time of the search (end of March 2024), a total of 38,316 publications were obtained, specifically: 7,282 in PubMed, 11,080 in Scopus, 17,117 in Web of Science, and 2,837 in ScienceDirect. The complete records were exported in.ris format and/or.csv format.

To facilitate the screening of articles, Rayyan [19] was used, a free tool specially dedicated to systematic reviews, which has an efficient interface for data management. In total, Rayyan recognized 38,312 publications.

### Screening

After importing all the references collected into Rayyan, potential duplicates were identified by considering main factors like title, authors, journal, and year. To speed up the process of elimination of duplicates, the Auto-Resolver was used, for those who had a confidentiality percentage greater than or equal to 95%. Those detected as a possible duplicate, but with a smaller percentage, were evaluated one by one. A total of 17,423 publications were removed, which is not surprising since the same publication can be in several databases. Although Rayyan automatically creates a list of inclusion and exclusion keywords, our own standardized list was defined by adapting it to our research objectives. Then, inclusion/exclusion filtering was carried out by considering the keywords in the title and abstract.

The entire paper was read for those publications where the filtering process remained uncertain. Subsequently, the full-text articles deemed suitable for eligibility were assessed for final inclusion.

**Fig. 1:**
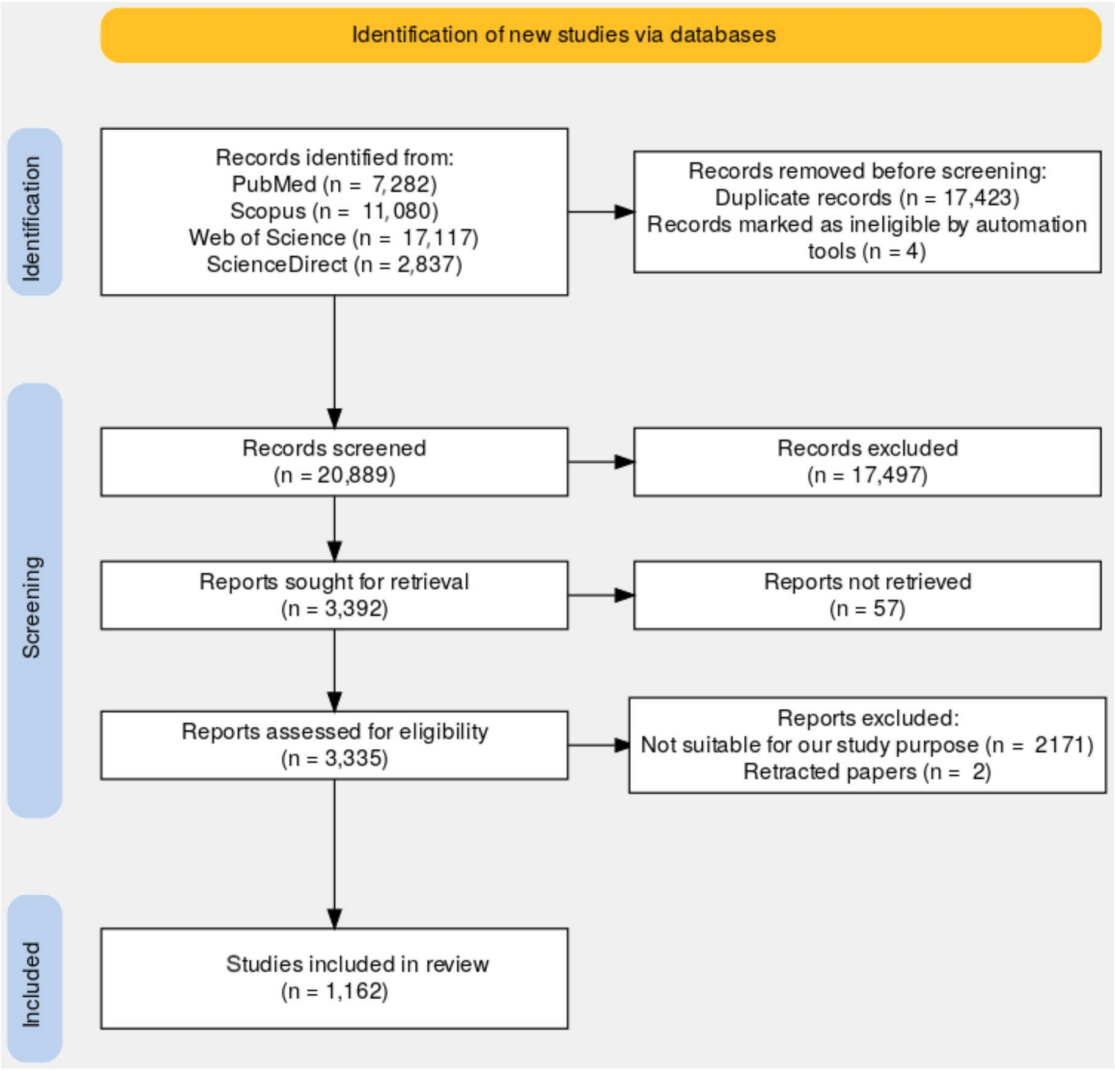
Database identification according to the PRISMA method [Flow chart for selecting publications, according to the PRISMA method]

The remaining 1,164 publications were imported into the free reference manager Zotero [20] to identify and exclude retracted papers. Zotero offers this functionality in collaboration with Retraction Watch [21], which maintains the largest available database of retracted articles. After this process, 1,162 articles were included in the final analysis (see Fig. 1 for more details) and can be consulted in the supplementary material.

### Data Adjustments and Management

When using more than one database, it is normal to find disambiguation in the data, since each source has a different format, so it is essential to check the.csv files. Not all publications provided included the number of citations, so the Zotero citation count manager was used [22], an add-on that auto-fetch citation counts for journal articles. On the other hand, the main metrics, such as the impact factor (JIF) and the quartile (Q) of scientific journals, did not appear in most of them, so the JCR (Journal Citation Reports) with Web of Science [23] was integrated.

Disambiguating author names is still one of the biggest challenges in bibliometrics [24]. Some discrepancies were easily resolved using the Python pandas library [25]. The more challenging problem involved identifying authors who published with different abbreviations that had similar names or shared common name structures, and it required manual intervention.

Due to the large number of publications, Python was applied because of its capability to manage extensive data and create clear visualizations. A total of 1,162 publications were exported in.csv format, due to their readability, scalability, and easy handling to integrate them with Python libraries necessary for our data analysis in bibliometrics and networks.

## Analyses and Results

### Bibliometric Analysis

Bibliometric research investigates authorship, publication patterns, citations, and content by applying quantitative measures to a selected body of literature [26].

To address this analysis, python libraries such as Pandas [25] for data manipulation and analysis, matplotlib [27] and seaborn [28] for visualization were employed.

- Scientific productivity

**Fig. 2:**
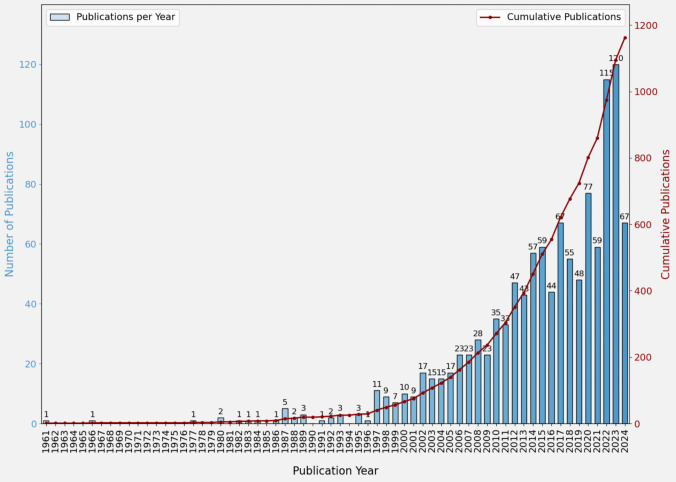
Publication Trends Over Time [Each bar in the graph represents the number of articles or studies published in each year. Each point in the graph represents the total number of publications that have been produced up to that year, adding up all the publications from previous years]

To evaluate the evolution and growth of literature in this field, a graph mapping the number of publications each year and the cumulative number of publications over time was generated (Fig. 2).

Research on the 'cerebellum in emotions' has grown significantly over time. Despite some minor fluctuations, interest in this field has consolidated and increased exponentially. Since 1997, publications have shown a steady increase, exceeding 100 per year as of 2022. This growth may be due to technological or methodological advances and new applications, with a marked acceleration phase coinciding with the discovery of cerebellar cognitive affective syndrome.[29].

**Fig. 3:**
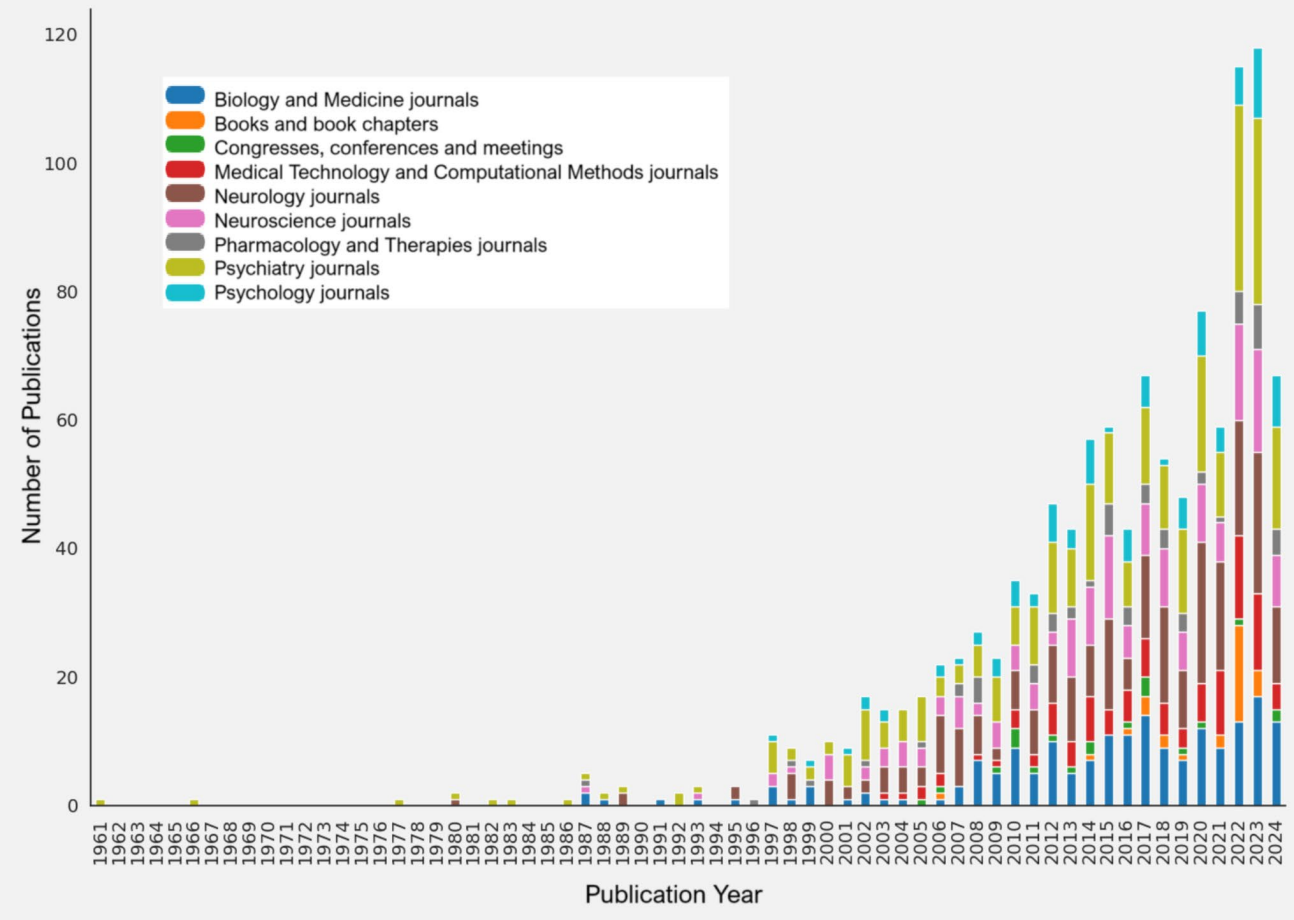
Annual Publications by Type [Each type/domain of publication is represented by a different colour]

To gain a deeper understanding of the evolution of research dissemination and to identify the dominant types of publications during specific periods, stacked charts were generated (Fig. 3), depicting the number of publications per year by publication type (e.g., Journals, Congresses, Books, etc.) and domain (e.g., Neuroscience, Pharmacology, etc.), as shown in Table S1.

Initial publications primarily focused on psychiatry, followed by neurology, biology, medicine, and neuroscience. These areas later expanded to include psychology, medical technologies, computational methods, pharmacology, and therapy, reflecting a progressive diversification. Scientific papers predominate over books and conference presentations, highlighted academic journals as the main means of dissemination.

As we mentioned in the previous figure and from a review of the titles and abstract, we note that the finding of the relationship between cerebellar damage and emotional and cognitive deficits, along with studies conducted in humans, prompted a successive wave of investigations that were consolidated by methodological refinements and technological advances.

- Journals

**Fig. 4:**
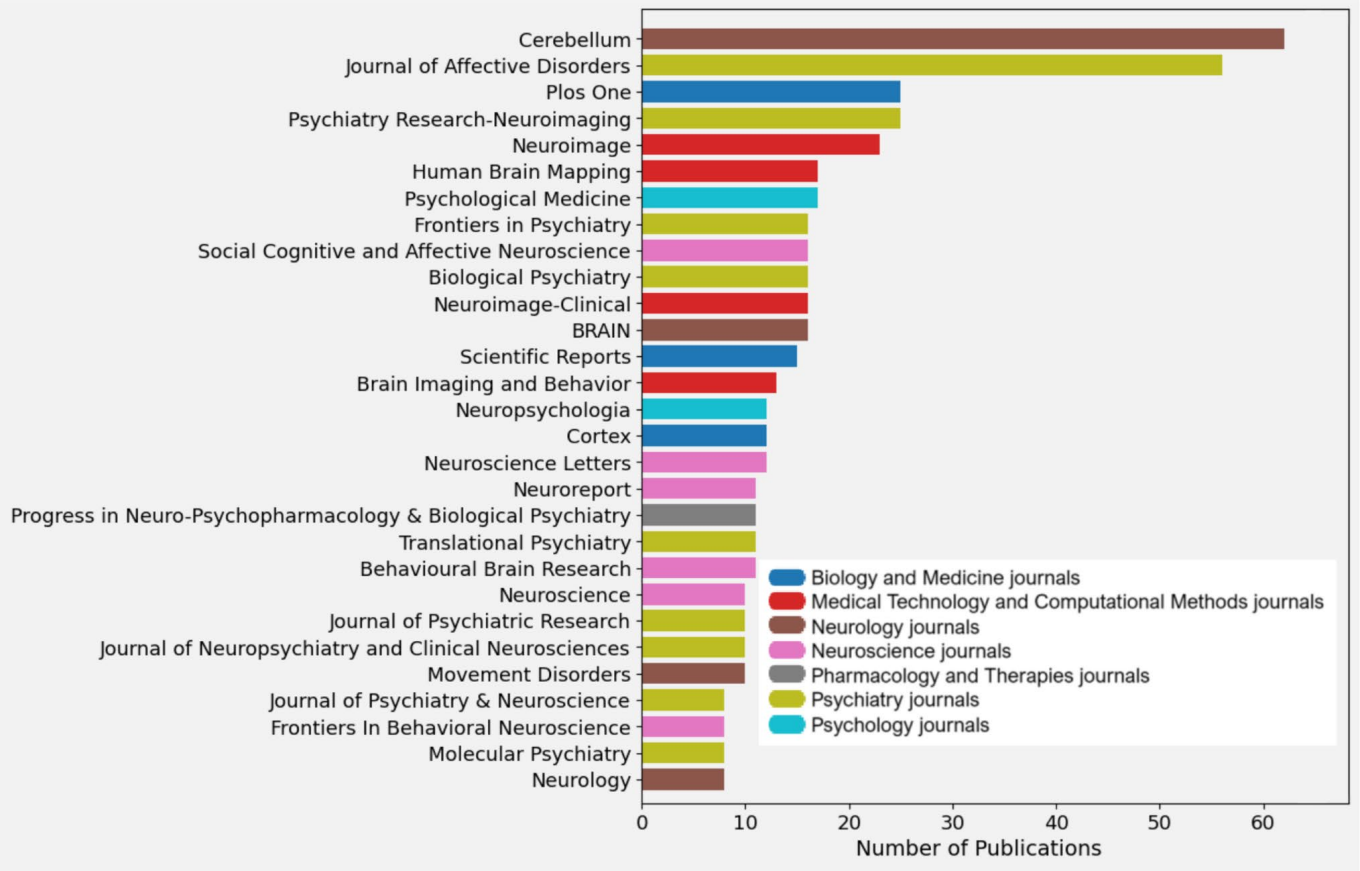
Leading Journals [The X-axis shows the number of publications and the Y-axis the name of the journal, the colour still codes the type/domain of papers]

Identifying the journals with the largest number of publications helps us understand where the most significant advances in the area are being shared and where to look for information. A bar chart (Fig. 4) represents the leading journals with their quantitative impact indicators and the type/domain of the publications on them. As an extension of this figure, a table (Table S2) presenting the impact factors and quartiles of each journal is included in the supplementary material.

Most publications are concentrated in journals focused on psychiatry, followed by neuroscience and neurology. The leading journals in this area are *The Cerebellum* [30], dedicated to cerebellar research and related disorders, and the *Journal of Affective Disorders* [31], which covers affective disorders, including depression, mood spectrum, emotions, anxiety, and stress.

- Citations

**Fig. 5:**
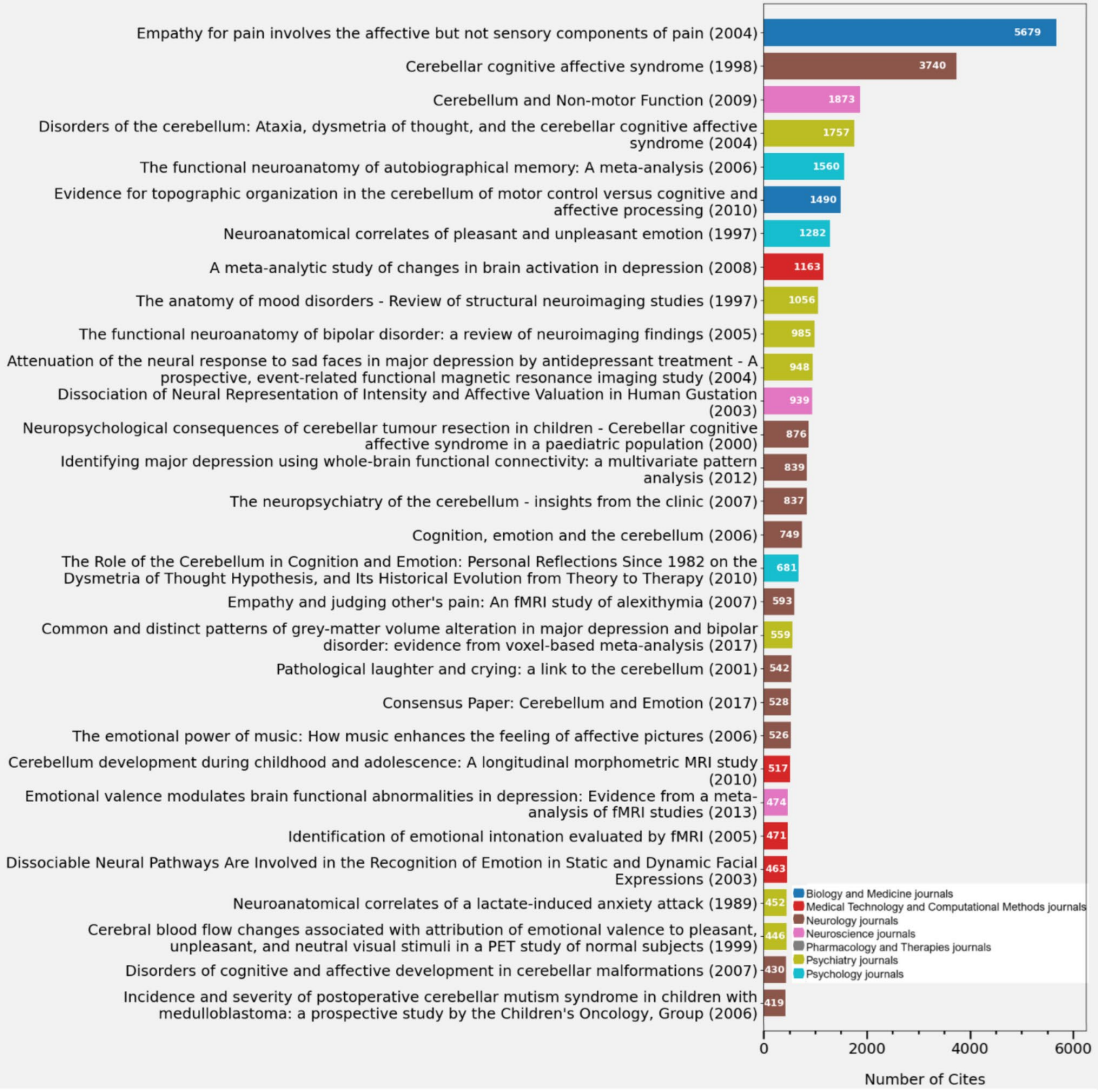
Leading cited publications [The Y-axis lists each research study along with its publication year, while the X-axis represents the number of citations received. Each bar is coloured according to the type of publications, making it easy to identify the most prolific and interesting areas]

Citations are an important indicator of the influence and impact of research. The most cited publications were selected and represented through a simple bar plot (Fig. 5). For more detailed information on the names of the journals in which each paper was published and the authors, see Table S3.

Two publications have a significantly greater impact with citations of 5,679 and 3,740, which is much higher than the others, starting to decrease from 1,873 citations onward. The first of them, published in 2004, although it is not the first to address the role of the cerebellum in the experience of pain, it is one of those that provide the clearest evidence about its activation in the context of empathy towards the pain of others. The second from 1998 on the cognitive-affective cerebellar syndrome was a key point in the recognition of the role of the cerebellum in emotions and marked a change in the traditional view of the cerebellum. Also, most of the highly cited publications are human imaging studies, with great relevance and impact, as they provide key information for the diagnosis, treatment, and understanding of diseases. This explains their high number of citations, given that findings in human imaging are often highly applicable both in research and clinical contexts.

- Keyword co-occurrence

Keyword co-occurrence analysis examines relationships and patterns between terms in a body of literature, visualizing their interconnectedness and revealing thematic structure. It allows identifying major themes, mapping knowledge networks, discovering promising or understudied areas, and focusing research on unexpected or less explored connections.

**Fig. 6:**
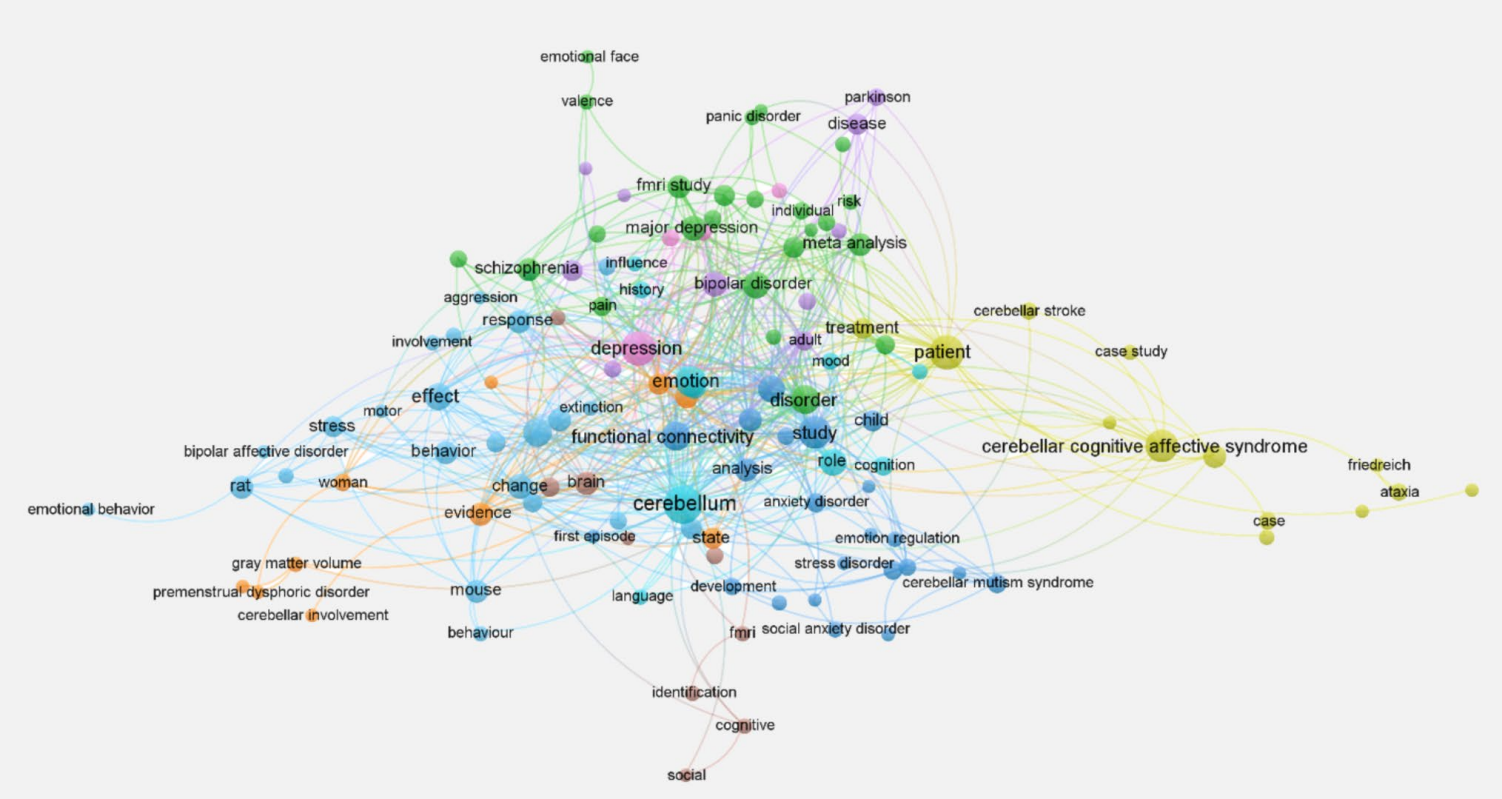
Keyword co-occurrence network [Each node represents a keyword; its size indicates its importance or frequency, while the lines show co-occurrences in the same context. Colors group semantic clusters linked to specific topics. The most central words in the network are near the center, indicating greater relevance, while peripheral words have less significant connections or deal with more specific topics]

For the co-occurrence analysis (Fig. 6), we considered the titles of scientific publications and used VOSviewer [32], a software tool specifically designed for the construction and visualization of bibliometric networks.

The word “cerebellum” is the main core of the corpus, and “emotion” one of the central nodes, which is not surprising given our search approach. Other relevant words (themes) are “functional connectivity”, “depression”, and “disorder”. There are subthemes connecting the cerebellum with specific conditions such as “major depression”, “schizophrenia” and “anxiety disorders”. One particular cluster is related to “cerebellar cognitive affective syndrome”, which links to other nodes such as “patient”, “ataxia” and “case study”. Other minor semantic clusters connect the cerebellum with cognitive and social functions, while another one links “behavior” with nodes such as “rat” and “mouse”, highlighting the study of the cerebellum in motor and emotional functions through animal models.

- Species

**Fig. 7:**
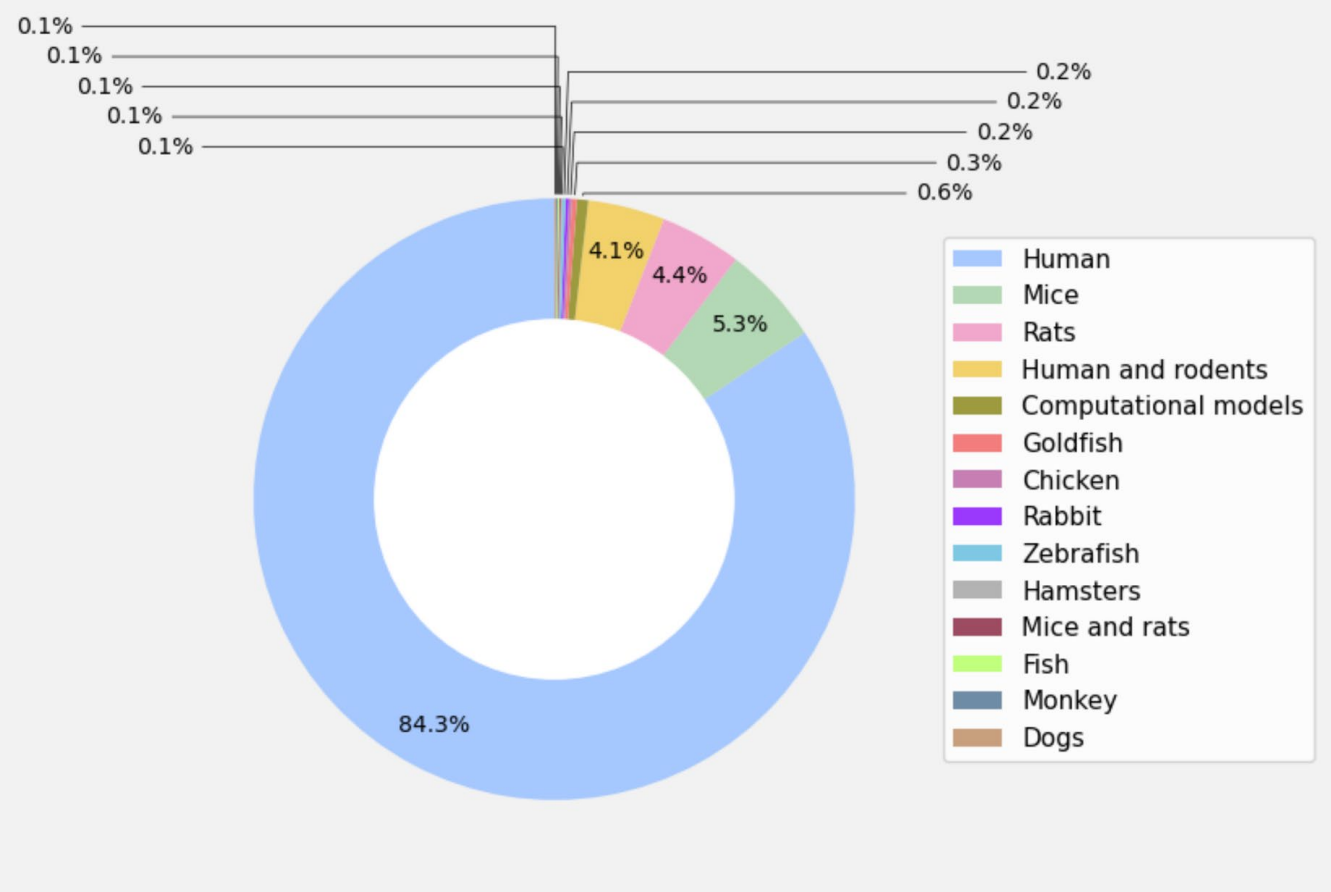
Distribution of species [Each segment of the pie chart represents the relative share of each species (including “modeling”) in the total]

Knowing which species have been investigated the most in the field and which have been investigated the least not only identifies trends and applicability of the studies, but also measures the diversity of approaches and methodologies, identifies possible biases in the research, and reflects economic or political influencing factors. A pie chart (Fig. 7) illustrates the distribution of species in the publications. Although computational modeling is not technically a species, it can be included in the analysis, since it represents a source of valuable in-silico data.

Human studies dominate research, reflecting a focus on medicine, psychiatry, and clinical applications. Rats and mice are the second and third most studied species, serving as essential models to explore biological processes and mechanisms underlying emotions, given their similarities to the human brain and practical advantages. Other species are minimally represented, emphasizing the translational focus on human applications.

Computational modeling, though promising, remains underdeveloped, comprising only 0.6% of studies. Existing models, such as the Emotional Cerebellar Model Articulation Controller (CMAC) and its variants, simulate cerebellar functions for complex system control, but further advancements are needed to match the depth of biological research.

- Topics

**Fig. 8:**
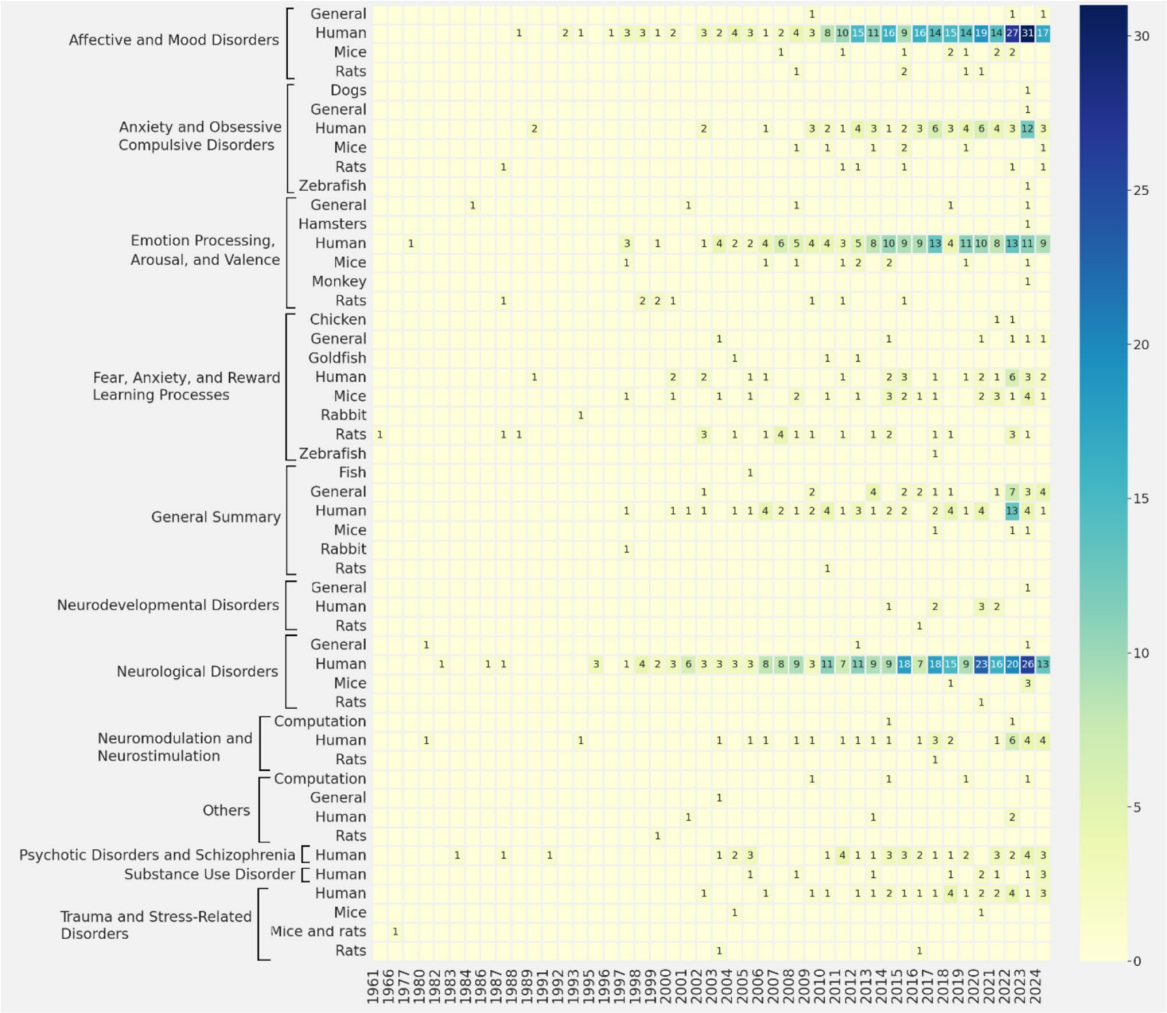
Thematic evolution by topic by species [Years on the horizontal axis, and topics and species on the vertical axis. Colours indicate the number of publications for each topic-species combination in a given year, and the colour gradient corresponds to the number of publications]

A deeper understanding of how research has been oriented over time combines both what is being investigated (topics) and on which species is conducted. First, the thematic category is identified; second, the thematic evolution by species is displayed, by a heat map (Fig. 8). Spikes in specific topics can indicate scientific breakthroughs, technological changes, or discoveries that have driven that topic forward.

**Fig. 9:**
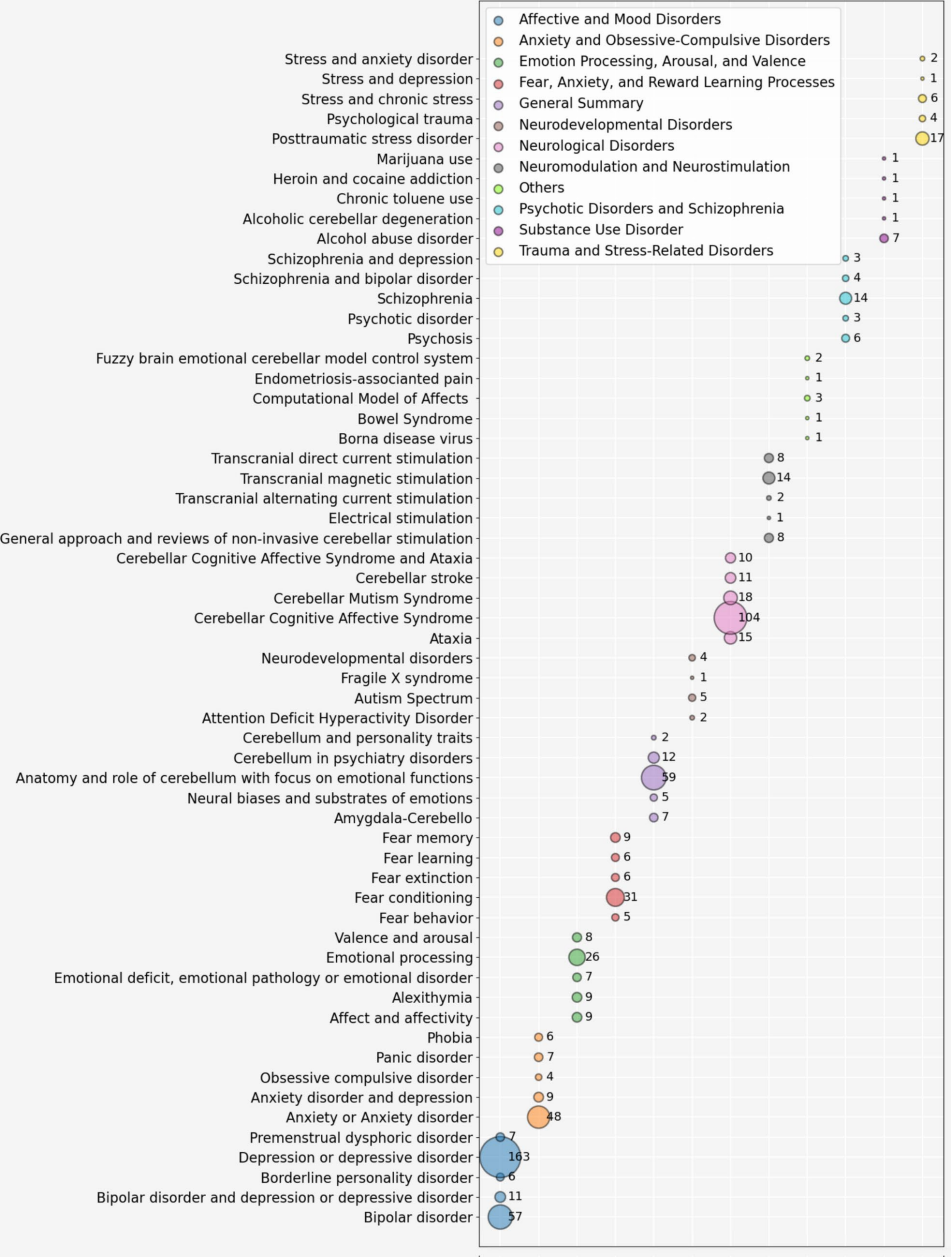
Leading topics [The X-axis displays the macro research areas, while the Y-axis represents specific topics within those areas. The size of each bubble indicates the number of publications related to that topic]

To offer an even more detailed view, a bubble chart shows the 5 most dominant topics in each area (Fig. 9).

Predominant topics include “neurological disorders”, “affective and mood disorders”, and “emotional processing, arousal, and valence” in humans. The least explored areas so far are “neurodevelopmental disorders”, “substance use disorder”, and “neuromodulation and neurostimulation”. In detail, in the area of “​​affective and mood disorders”, human studies predominate, with a significant increase starting in 2010 and a peak starting in 2022. To a lesser extent, there were publications on this topic in mice and rats, whose studies began in 2007 but without continuity. In the area of ​​ “anxiety and obsessive–compulsive disorders”, publications in humans also predominate, with interest emerging since 2009 and peaking in 2023. Regarding “​​emotional processing, arousal, and valence”, studies in humans have been constant since 2001, showing an increase since 2013. There are also publications in mice and rats, which are smaller and less frequent. In the area related to “fear, anxiety, and reward learning processes”, research is more evenly distributed between humans, mice, and rats, with a slight predominance in humans. In the area we call “general summary”, the focus on humans stands out, with a peak in 2022, followed by those writings where more than one species are mentioned (General). “Neurodevelopmental disorders” are a relatively under-researched topic, and focus especially on humans. Most studies focus on “neurological disorders”, which have seen steady growth since 2006, with a notable peak in 2020. Publications involving rodents in this area are quite limited. Additionally, fields such as “neuromodulation and neurostimulation”, as well as “trauma- and stress-related disorders”, also show a clear preference for human studies. Finally, research on “psychotic disorders and schizophrenia”, and “substance use disorders” has been conducted exclusively on humans.

- Main techniques used in the research

The analysis of the techniques employed in the investigation of the cerebellum and its role in emotions provides a comprehensive and simplified view of a complex picture. This helps to highlight trends, identify the most researched areas and reveal possible gaps in the use of methodologies, which is essential to guide future research.

**Fig. 10:**
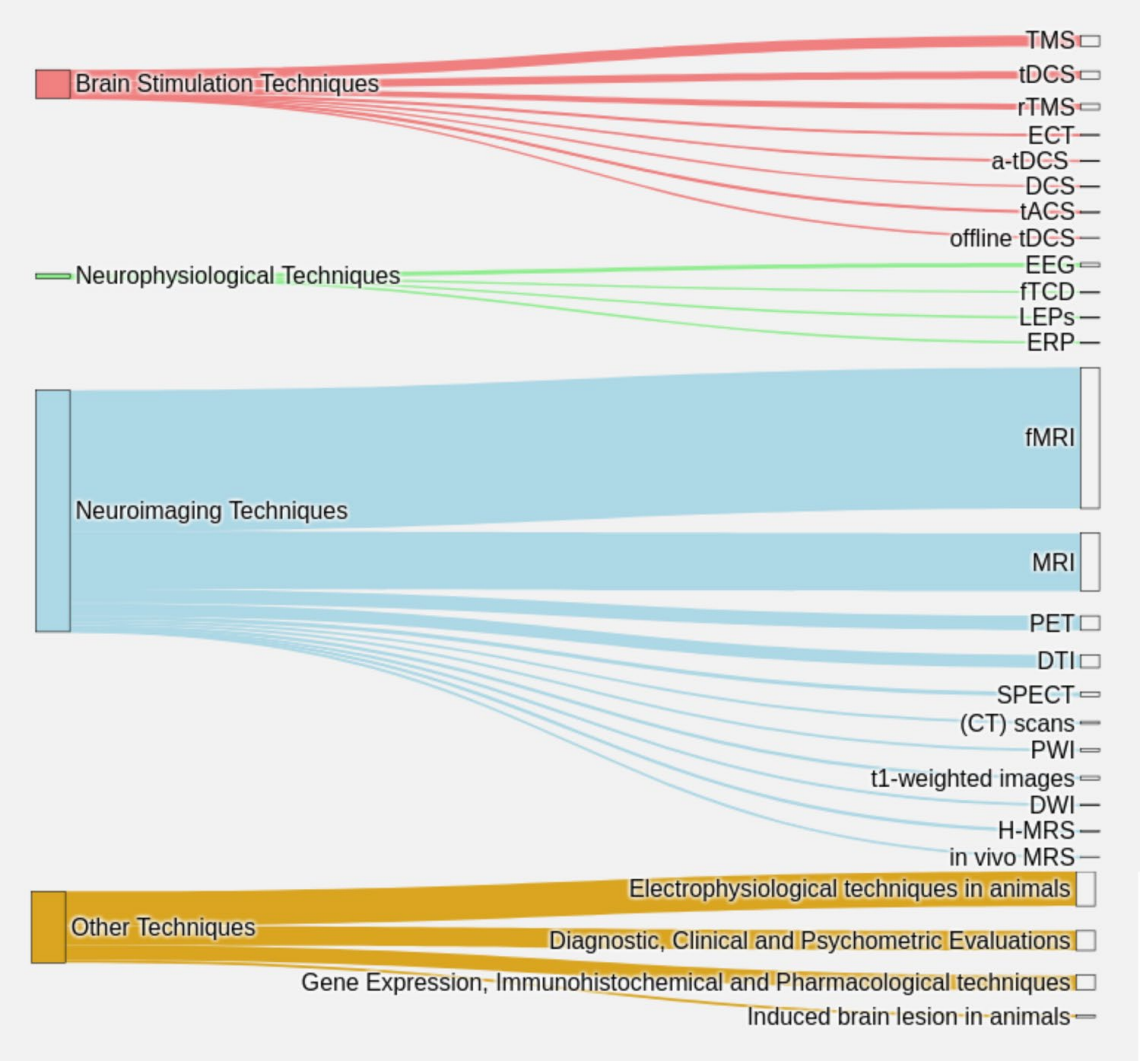
Techniques of research [The thickness of the lines indicates the frequency of use of each technique in the studies analyzed. Main Categories: Brain Stimulation Techniques: Includes methods such as rTMS (Repetitive Transcranial Magnetic Stimulation), ECT (Electroconvulsive Therapy), a-tDCS (Anodal Transcranial Direct Current Stimulation), DCS (Direct Current Stimulation), tACS (Transcranial Alternating Current Stimulation), Offline tDCS (Offline Transcranial Direct Current Stimulation), which are used to stimulate or modulate brain activity. Neurophysiological Techniques: include EEG (Electroencephalography), fTCD (Functional Transcranial Doppler), LEPs (Laser-Evoked Potentials), ERP (Event-Related Potentials) methods, which measure brain activity. Neuroimaging Techniques: Comprises advanced methods such as fMRI (Functional Magnetic Resonance Imaging), MRI (Magnetic Resonance Imaging), PET (Positron Emission Tomography), DTI (Diffusion Tensor Imaging), SPECT (Single Photon Emission Computed Tomography), (CT) Scans (Computed Tomography Scans), PWI (Perfusion-Weighted Imaging), T1-weighted Images, DWI (Diffusion-Weighted Imaging), H-MRS (Proton Magnetic Resonance Spectroscopy), In vivo MRS (In vivo Magnetic Resonance Spectroscopy), used to study structures, functions and biochemical processes in the brain. Other techniques include experimental and analytical tools in both animals and humans]

For this analysis, we used a Sankey diagram (Fig. 10), which effectively organizes and displays the connections between categories.

Neuroimaging techniques stand out as essential tools in research because of their noninvasive capacity to analyze brain activity and structure, with emphasis on the interaction of the cerebellum and emotional regions. Although stimulation and neurophysiological methods complement this exploration by modulating and evaluating cerebellar activity in real time, their use is secondary. On the other hand, animal models and preliminary techniques are of limited use, suggesting an approach mostly applied to humans, consistent with the complexity of emotions.

- Researchers

In a bibliometric analysis, identifying the authors with the highest number of publications in a specific area of interest is essential. It helps pinpoint the most influential and active researchers, who often drive advancements and set trends within the field.

Additionally, these authors are frequently part of key collaboration networks, making it easier to recognize scientific communities and their working dynamics. It also reveals which areas or topics an author publishes in most, allowing for insights into their expertise and research focus.

**Fig. 11:**
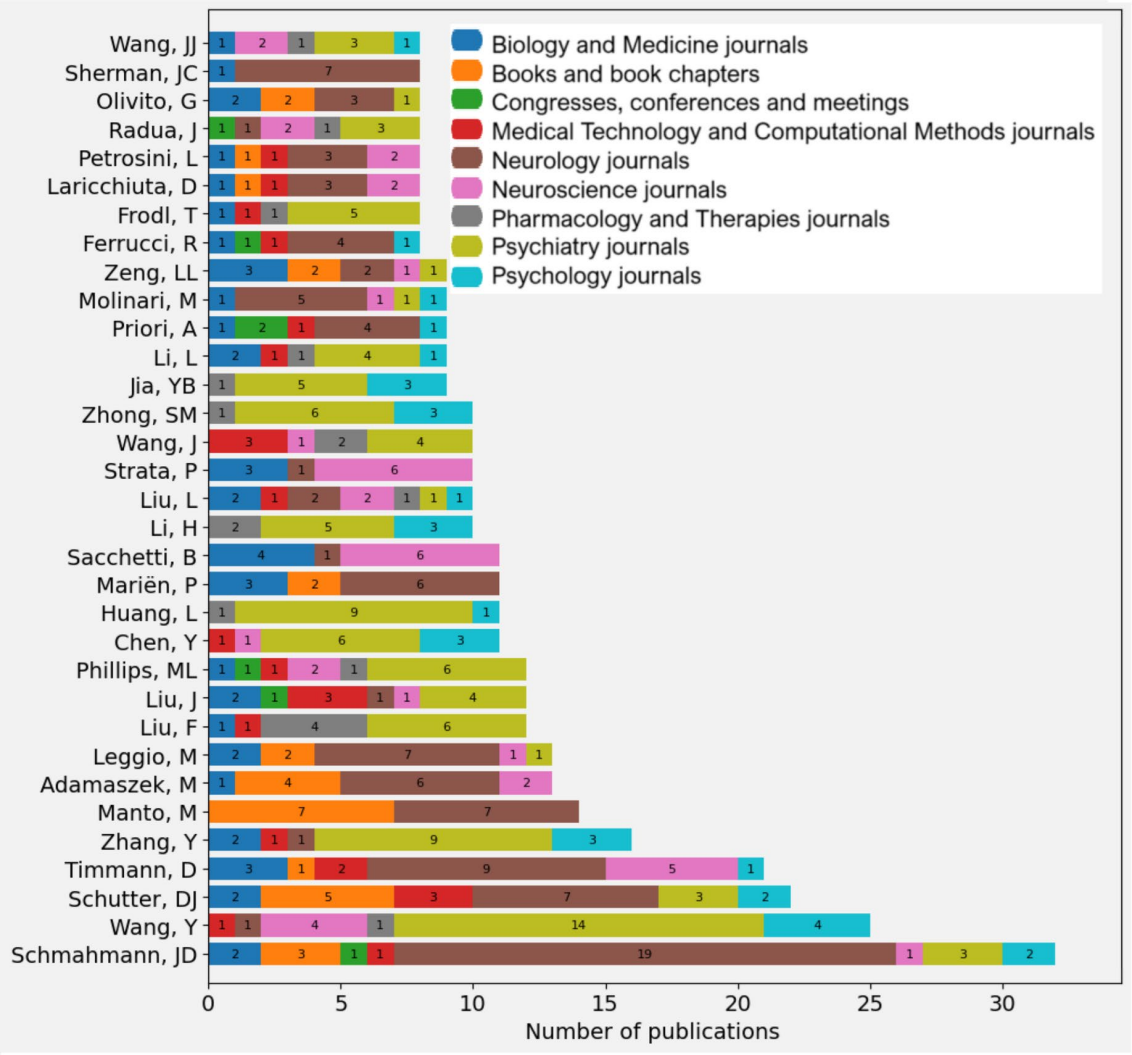
Leading authors [Each bar represents an author and is divided into colouring segments indicating the number of publications according to his/her type/domain of publication. The X-axis shows the number of publications, while the Y-axis shows the names of the authors]

A stacked bar chart (Fig. 11) shows the ranking of authors, assigning equal weight to all to reflect collective contributions without specific hierarchies.

It is known that, at times, the position of authors in a publication may reflect the roles they played in the project (study leader, key support in analysis, experiments or knowledge, supervisor, project manager, and funding). This aspect, detailed in Figures S1 and S2, helps to clarify the contributions and leadership of each author, offering a perspective on their academic impact.

The most prolific and influential author on the subject is Schmahmann, with a greater number of publications in Neurology, which is not surprising since he is known for his pioneering work [33] on the role of the cerebellum in cognition and emotion, and the description of the cerebellar cognitive-affective syndrome (also called Schmahmann syndrome). The five authors with the highest number of publications are Schmahmann, with more than 30 publications; Wang, with 25 publications; Schutter and Timmann, with more than 20 publications each; and Zhang, with 16 publications. This indicates a high level of research activity. In general, most authors have between 5 and 15 publications. It is observed that researchers publish in journals of different related topics, which suggests a trend toward interdisciplinarity.

- Patterns of Collaborations

**Fig. 12:**
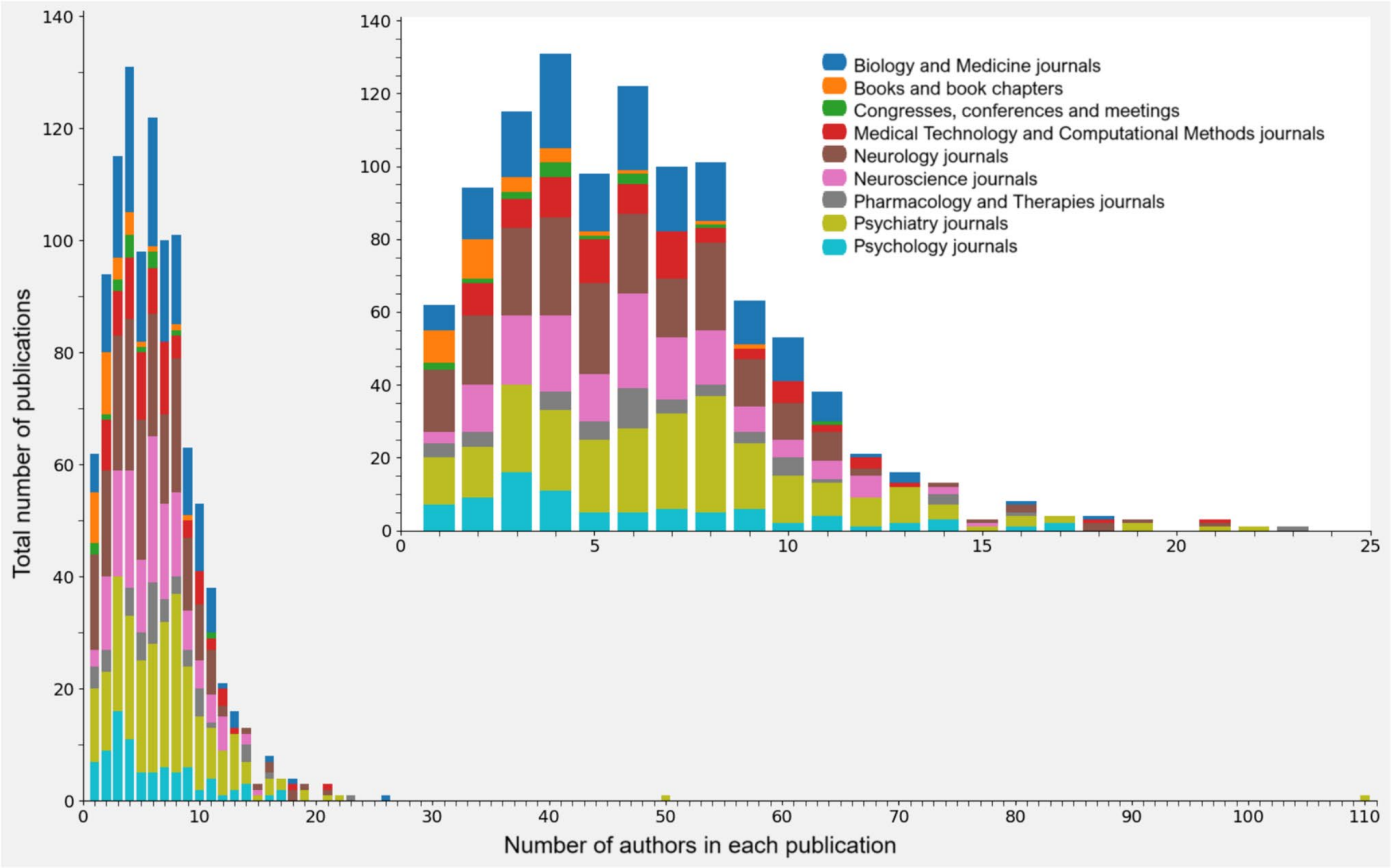
Distribution of Publications by Author Count [Distribution of publications based on the number of authors, with stacked bars differentiating between type/domain of publications]

**Fig. 13:**
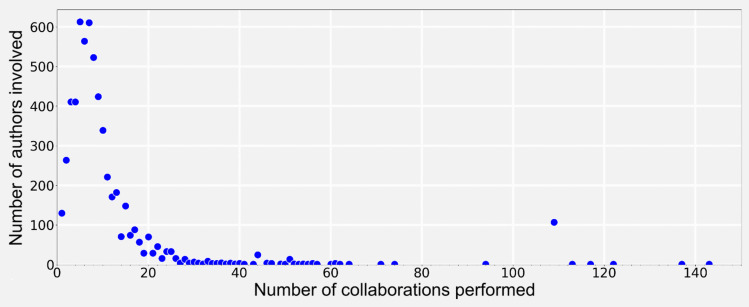
Collaboration Patterns by Number of Authors [Scatter plot of the number of collaborations per author. It displays individual collaboration levels]

To understand how knowledge is produced in this field, patterns of collaborations and authorships can be described, specifically how researchers collaborate on publications, focusing on the number of authors involved and the frequency of their contributions across multiple works (Figs. 12 and 13). Figure 12 shows how the total number of publications varies according to the number of authors involved in each publication. On the other hand, Fig. 13 illustrates how many authors are involved in different numbers of collaborations, focusing on the frequency of their collaborative activity.

Most of the publications have between two and eight authors, with a peak of four. Only two publications stand out for their high number of authors (50 and 110) in the field of clinical research. These high-author counts are attributed to multi-center studies, diversity of specializations, large clinical trials, and international collaborations. The prevalence of publications with fewer authors suggests that limited collaborations may enhance specialization in specific research topics.

The majority of authors have few collaborations, as collaborations increase, the number of authors decreases exponentially, forming a “long tail”.This tail represents a small group of highly prolific authors, some with over 100 collaborations. This suggests a collaborative structure where a core group of authors frequently contributes to multiple projects, while most participate occasionally. Additionally, these collaborations involve various combinations of authors rather than fixed teams.

- Funding

Analyzing the sources of funding sheds light on the resources available to researchers and the priorities of funding agencies, evaluates the relationship between investment and scientific impact, facilitates strategic collaborations, and helps in planning for future investments in research and development. However, it is important to note that not all research articles report funding due to various reasons, such as the use of their own resources, standard institutional infrastructure, or public data that do not require explicit funding. Also, not all journals have strict policies on funding disclosure, mainly in older papers. In other cases, funding may be indirect, coming from previous projects, institutional resources, or general grants not specifically assigned to the work in question.

Table 2 shows the public and private organizations that have funded research on the cerebellum and its role in emotions, based on the information reported in the scientific publications.

**Table 2:** Funding Organizations^***^

Private
Alexander von Humboldt Foundation, DE Alzheimer's Association, US Baasch-Medicus Foundation, CH Baylor College of Medicine (BCM), US BCM-Intellectual and Developmental Disabilities Research Center (IDDRC), US Béatrice Ederer-Weber Stiftung, CH Birmingham Foundation, US Brain & Behavior Research Foundation (BBRF), US BBRF-Young Investigator Grant, US BBRF- National Association for Research on Schizophrenia and Depression (NARSAD),US Brain Research Foundation (BRF), US Davis Fellowships, US Deutsche Stiftung Neurologie (DSN), DE Erasmus MC Foundation, NL Else Kröner-Fresenius-Stiftung, DE Fondation Leenaards, CH Fondation pour la Recherche Medicale, FR Friedreich's Ataxia Research Alliance, US Fund for Medical Discovery (FMD) Clinical Research Fellowship Award,US Hamill Foundation, US Heidehof Stiftung, DE Hertie-Stiftung, DE Instrumentarium Science Foundation, FI James S.McDonnell Foundation, US James McKeen Cattell Fund, US La Caixa Foundation, ES Max Planck Society, DE MINDLink Foundation, US National Ataxia Foundation, US Physiological Society, UK Physiological Society-Undergraduate training in in vivo sciences grant, UK Physiological Society-Vacation Scholarship awarded, UK Sigrid Juselius Foundation, FI Swiss National Science Foundation (SNSF), CH Texas Children’s Hospital, US The Ataxia Telangiectasia Children’s Project, US The Berthold Leibinger Stiftung GmbH, DE The Paulo Foundation, FI The Phyllis & Jerome Lyle Rappaport Foundation, US The Reinhold Beitlich Foundation, DE The Synapsis Foundation, CH The Finnish Medical Foundation, FI Wellcome Trust, UK Wellcome Trus-Sir Henry Wellcome Postdoctoral Fellowships, Sir Henry Dale Fellowships, UK Whitehall Foundation, US Wyss Foundation, US
**Public**
Agence Nationale de la Recherche (ANR), FR ANR, CerebellEMO, FR Australian Research Council (ARC), AU ARC-Australian Laureate Fellowship, Centre of Excellence for Integrative Brain Function, Centre of Excellence in Cognition and its Disorders, Discovery Early Career Researcher, Science of Learning Research Centre, AU Australian Government, AU Research Training Program (RTP) Scholarship, Primary health and medical research funding agency, AU Austrian Science Fund (FWF), AU Beijing Municipal Science & Technology Commission Grant, CN Biotechnology and Biological Sciences Research Council (BBSRC), UK Canadian Institutes of Health Research (CIHR), CA Consejo Nacional de Investigaciones Científicas y Técnicas, AR Consejo Nacional de Ciencia y Tecnología (CONACYT), MX Consejo Superior de Investigaciones Científicas (CSIC), ES Conselho Nacional de Desenvolvimento Científico e Tecnológico, BR Deutsche Forschungsgemeinschaft (DFG), DE Dutch Ministry of Health, NL Department of Health and Human Services (HHS), US European Molecular Biology Laboratory (EMBL) European Molecular Biology Conference (EMBC) EMBC- European Molecular Biology Organization (EMBO) European Regional Development Fund (FEDER) grant European Research Council (ERC) ERC-Horizon 2020 (H2020), Seventh Framework Programme (FP7), Starting Grant H2020- ActionContraThreat, NeuroTRACK, REALNET, LTFCOFUND2013, Human Brain Project (HBP), Cerebellum and Emotional Networks (CEN) FP7, CEREBNET Foundation for Dutch Scientific Research (NWO), NL NOW-Veni grant, NL Fondazione IRCCS, IT Fundaçao de Amparo à Pesquisa do Estado de São Paulo, BR Fundação para a Ciência e a Tecnologia, PT Guangdong Basic and Applied Basic Research Foundation,CN Grants-in-Aid for Scientific Research (KAKENHI), JP Institut National de la Santé et de la Recherche Médicale, FR Italian Ministry of Health, IT Japan Agency for Medical Research and Development, JP Japan Society for the Promotion of Science (JSPS), JP Korea Science and Engineering Foundation (KOSEF), KR Labex Memolife, FR Ministry of Education, Universities and Research, IT Ministry of Education, Universities and Research, MNESYS, IT Ministry of Health, Welfare and Sport, NL Ministry of Science and Technology (MOST), TW Munich University Hospital of the Ludwig-Maximilians-Universität München (LMU),DE National Basic Research Program of China, CN National Institute of Health Carlos III (ISCIII), ES ISCIII-Miguel Servet contract, ES National Key Research and Development Program of China, CN National Natural Science Foundation of China, CN National Research Foundation of Korea (NRF), KR NRF- KOSEF grant, KR National Science Foundation CAREER award, US21 Natural Science Foundation of Hunan Province, CN National Competence Center for Research SYNAPSY, CH National Institute of Neurological Disorders and Stroke, US National Institutes of Health (NIH), US NIH-National Institutes of Neurological Disorders and Stroke (NINDS), National Institute of Nursing Research (NINR), National Cancer Institute (NCI), National Institute on Aging (NIA), National Center for Advancing Translational Sciences (NCATS), National Center for Complementary and Integrative Health (NCCIH), National Institute on Alcohol Abuse and Alcoholism (NIAAA), National Institute of Biomedical Imaging and Bioengineering (NIBIB), National Institute on Drug Abuse (NIDA), NIH, National Institute of Environmental Health Sciences (NIEHS), National Institute of Child Health and Human Development (NICHD), National Center for Research Resources (NCRR), National Institute of General Medical Sciences (NIGMS), National Institute of Mental Health (NIMH), US Natural Sciences and Engineering Research Council of Canada (NSERC), CA Nederlandse Organisatie voor Wetenschappelijk Onderzoek, NL Projekt DEAL, DE Russian Science Foundation, RU Rutherford Discovery Research Fellowship, NZ Shandong Provincial Natural Science Foundation, CN Strategic Basic Researc funding programme (SBO), BE UK Research and Innovation (UKRI), UK UKRI- Medical Research Council (MRC), UKC Veterans Affairs (VA), US VA-Clinical Science Research and Development (CSRD), US West China Medical School and West China Hospital, CN 21st Century Frontier R&D Program, Korea Brain Korea 21 Program for Leading Universities & Students, Korea

^***^ The classification between public and private organizations is not strict. It is important to mention that, although a careful categorization has been attempted, the classification is an approximation that seeks to improve the understanding and analysis of the information obtained from the scientific articles

Financial support from diverse funding bodies including government agencies, ministries, national research councils, associations, foundations, and independent public and private organizations, are supporting this research field. Particularly in the U.S., Europe, Australia, the U.K., and China. However, limited funding in Latin America, Africa, and parts of Asia highlights disparities in resources and infrastructure, restricting participation in this field.

- Affiliations

**Fig. 14:**
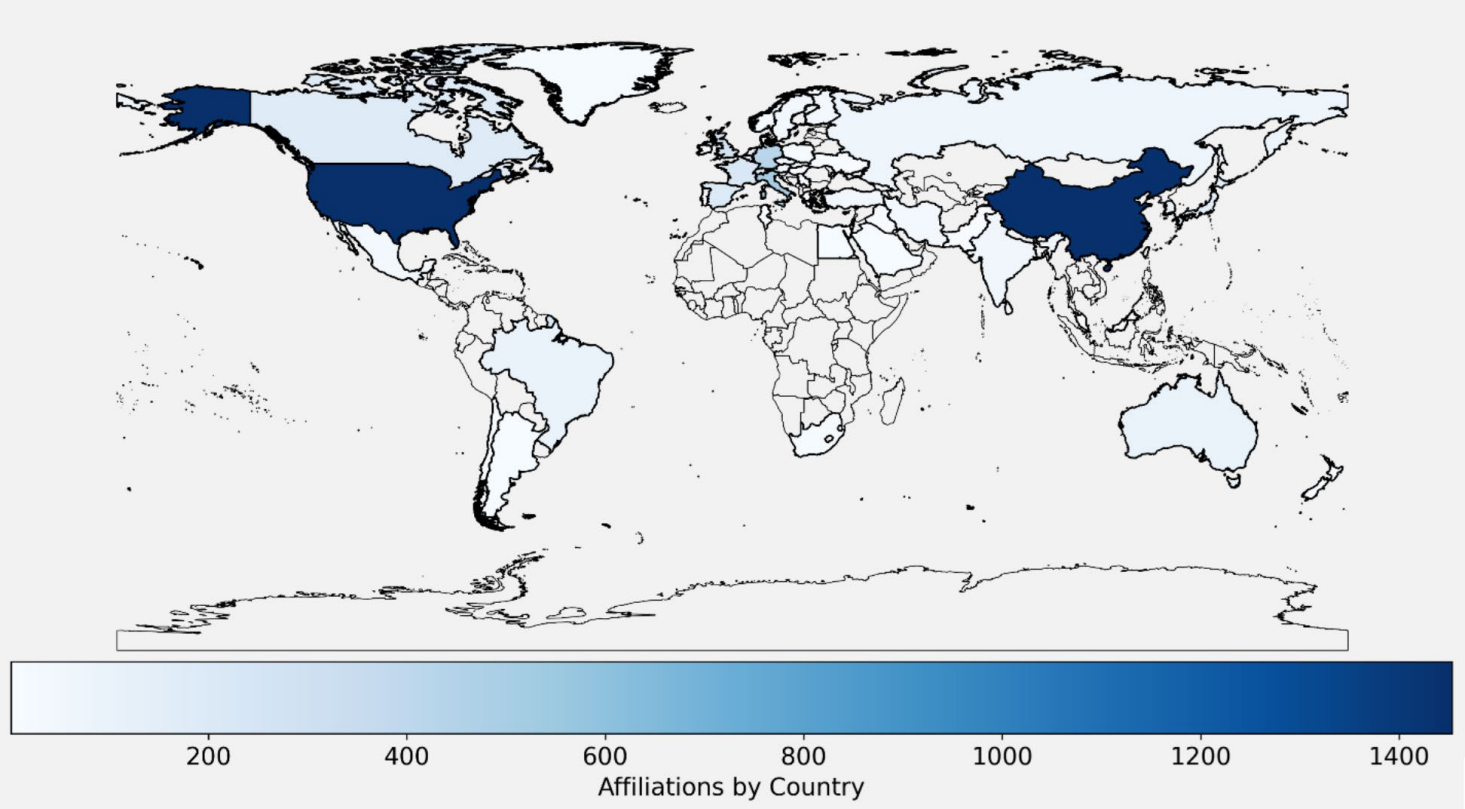
Geographic Distribution of Authors' Affiliations [A colour gradation identifies leading and less-developed regions in terms of involvement in this scientific research]

Author affiliation plays a key role in scientific production, reflecting the resources and infrastructure of institutions rather than the researchers' nationality. Understanding affiliations help identify leading countries and institutions, reveal regional inequalities, guide global collaboration, and inform funding priorities. A world map (Fig. 14) combines geographic and scientific data to illustrate these dynamics created using GeoPandas [34].

Author's affiliations show significant regional disparities. The U.S. and mainland China lead in publications, followed by some European countries, the U.K., and Canada, supported by strong infrastructure, technology, and funding (observed in Table 2). Countries like Australia, Japan, South Korea, Brazil, and Mexico rank at an intermediate-low level, while regions/economies such as Taiwan (China), Hong Kong (China), Israel, Singapore, India, Argentina, Chile, South Africa, Russia, Saudi Arabia, and Iran show very low output. Africa, more Latin American countries, and Central Asia are largely underrepresented due to limited infrastructure, funding (observed in Table 2), and prioritization of research, further exacerbated by the "brain drain" and difficulties in gaining global recognition.

### Collaboration Networks

This section focuses on the temporal analysis of academic collaboration networks and co-authorship dynamics. This analysis exploits two Python libraries designed for the creation, manipulation, and study of complex network structures, NetworkX [35] for exploratory analysis and the calculation of metrics, and Graph-tool [36] for large-scale network visualization.

- Visualization of the Collaboration Network

**Fig. 15:**
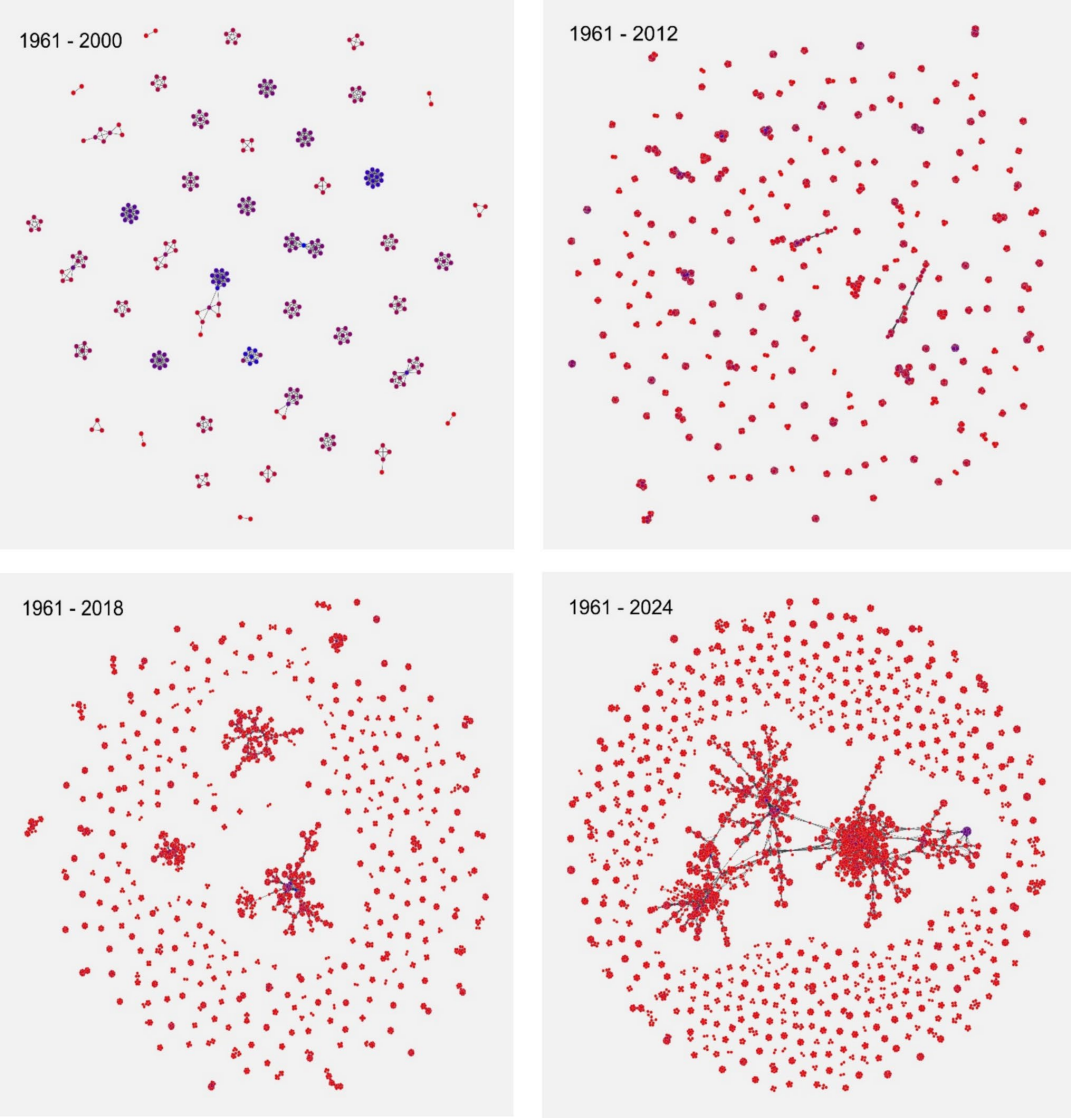
Evolution of the Collaboration Network in Different Periods of Time [Evolution of the network in four periods. Nodes are colored and scaled based on their degree centrality, which reflects the number of connections each node has. Highly connected nodes are blue, peripheral ones with fewer connections are red, and intermediate nodes are purple. The layout minimizes overlap, clustering more connected nodes together. Duplicate edges are removed to avoid clutter, and "bunches of grapes" represent clusters or communities of closely connected nodes]

A network graph is useful to understand a complex system and how its components interact with each other [37]. In a collaboration network, the nodes represent individual entities (authors), while the connections indicate collaborative relationships between them in scientific publications. The Scalable Force-Directed Placement algorithm available in the NetworkX library [35] provides a clear and scalable visualization of the graph structure over different periods. Figure 15 illustrates how the network has evolved, based on four selected periods, allowing us to assess how collaborations develop and change throughout these intervals.

In the first period 1961–2000 (top left image) the network is small, consisting of compact, isolated groups with limited connections. Key nodes (in purple blue) serve as links between groups, indicating preliminary alliances.

During the period 1961–2012 (top right image), a significant increase in nodes and connections is observed, leading to a dispersed network with no dominant nodes or groups. Clusters operate autonomously, with strong internal connections but limited interactions between clusters, except for a few bridging groups.

Between 1961–2018 (bottom left image), the network grows further, with higher connection density and reduced isolation of peripheral clusters. Emerging hubs act as central nodes, fostering inter-group collaboration and reflecting a growing number of collaborators.

The complete network, spanning from 1961–2024 (bottom right image) achieves high complexity, with numerous nodes and dense clusters. Subgroups are clearly visible, indicating frequent collaborations within areas of expertise or specialized teams. The absence of a dominating node highlights a balanced, distributed structure. Network.

- Metrics Analysis

The metrics used in graph theory [37–40] not only enhance visualizations by providing concrete data but are also essential for characterizing and analyzing the collaboration network, offering a deeper understanding of its structure, key nodes, and temporal dynamics.

We strongly encourage readers to review [37–40] for theoretical foundation, as well as the Supplementary Table S4 which presents a detailed analysis and explanations of the network's main metrics, assessing its structure and behavior over time. This will provide a better understanding of the analysis.

The metrics confirm quantitively the previous observations in Fig. 15. There is significant growth in the number of publications, researchers and collaborations, reflecting increased scientific activity and interaction among participants. In addition, the network is becoming more cohesive, with larger groups of people working together and an increase in the formation of small, well-connected teams, which act as “hubs” for collaboration in specific areas.

When analyzing the most important connections within the network, it is observed that, although researchers are more distributed and less concentrated in one place, some of them become key intermediaries to connect different areas of knowledge. There is also a general improvement in connectivity among participants, which means that it is easier for anyone within the network to access information or collaborate with others.

Diversity in connections increases, indicating that researchers are collaborating with more people and on more varied topics. At the same time, small groups that used to be very isolated are becoming more integrated, forming a more interconnected network. However, while the network is more resilient to the loss of random connections, it is still vulnerable if certain key individuals or teams that act as important bridges are lost.

Overall, the network not only grows in size, but also becomes more complex, diverse and integrated. This reflects more efficient scientific collaboration, with broader interactions and a structure that fosters both specialization and connection between different areas of knowledge.

## Discussion

Advances in the field**:** Research on the cerebellum's role in emotions has expanded significantly, driven by the identification of cerebellar affective-cognitive syndrome, technological advancements and interdisciplinary approaches. Early research focused primarily on psychiatry and neurology, but the field has since expanded to incorporate disciplines such as psychology, medical technologies, computational methods, pharmacology, and therapeutic strategies. These developments have deepened our understanding of the cerebellum's impact on emotional processing particularly in the context of neurological and affective disorders.

Methodological and thematic trends: Neuroimaging techniques have become indispensable due to their noninvasive capacity to analyze brain activity and structure, particularly in studying the interactions between the cerebellum and emotional regions. While stimulation and neurophysiological methods provide complementary insights, their application remains secondary. Research is predominantly focused on human studies, underscoring their clinical relevance and importance to medical practice. In contrast, animal models, such as mice and rats, are underrepresented, with limited exploration of other species. Similarly, computational models, although promising, are still in their early stages, emphasizing the need for further development of multiscale models [49–51] that integrate structural, functional, and dynamic aspects across multiple levels—from micro to macro scales—will be pivotal in advancing our understanding of its multidimensional functions. These models should leverage insights from experimental studies, computational modeling, and clinical research to construct a comprehensive framework for exploring the cerebellum’s complexity and its interactions within broader neural networks.

Evolution of collaboration scientific networks: The analysis of collaboration networks reveals a balance between specialization and interdisciplinarity. These networks are characterized by large nodes or "clusters," which represent influential authors or highly productive teams, alongside numerous peripheral nodes that reflect less integrated participants. While these decentralized structures foster innovation and resilience, challenges related to global connectivity and integration remain. Efforts should focus on strengthening global connectivity in collaborative networks by promoting international funding and open-access initiatives. Encouraging deeper interdisciplinarity, particularly with emerging fields like artificial intelligence and medical technologies, could drive innovation. Supporting less integrated participants and leveraging advanced computational tools for network analysis may further enhance collaboration and scientific impact.

Comparison with other research areas: The evolution of research on the cerebellar role in emotions shares similarities with other fields in terms of exponential growth, although the driving factors vary. For example, although with a higher volume of publications, in areas such as resting-state fMRI research on schizophrenia [41] growth also started slowly but has increased considerably reflecting a growing interest. In contrast, fields like artificial intelligence applied to radiation therapy [42], virtual reality for addressing depression and anxiety [43], and deep learning in cancer [44] present a growth starting much later and abruptly and explosively in a very short time. On the contrary in areas such as emotional intelligence and social interaction [45] exhibit lower but steady production, with occasional fluctuations.

Regional challenges and inequalities: Global research output tends to be concentrated in high-income areas with strong resources and infrastructure, while other regions face challenges such as limited funding, "brain drain," and lower visibility in the scientific community. These disparities highlight the importance of fostering international collaborations, increasing public and private investment in research, and enhancing access to resources in underrepresented areas. Encouraging the use of open-access journals and reducing or eliminating publication fees can also contribute to greater equity in knowledge dissemination and visibility.

Philosophical perspective, evolution of the paradigm: Traditionally, the cerebellum was considered a structure dedicated exclusively to motor control, and research for decades adhered to this paradigm. However, evidence of its involvement in emotional and cognitive functions has challenged this view, prompting a significant revision of the paradigm. Rather than discarding the previous framework, these discoveries have enriched and reconfigured scientific understanding, revealing a more complex and multidimensional role for the cerebellum. According to Thomas S. Kuhn [46], a scientific revolution involves the replacement of a dominant paradigm when anomalies accumulate and cannot be explained within the existing framework. In the case of the cerebellum, although anomalies have emerged, they have not led to a paradigm shift or scientific revolution. Instead, this represents a refinement of the existing paradigm. Other philosophical perspectives offer complementary insights. Imre Lakatos’ theory of research programs [47] might interpret these advancements as a progressive transition within the current framework of cerebellar studies. Similarly, Willard Van Orman Quine’s view of science as a continuous, interconnected system of beliefs [48] could frame this as an adjustment within the broader network of scientific knowledge, rather than a radical transformation.

## Conclusions

This study provides a thorough and updated analysis of the cerebellum's role in emotions, using a bibliometric and collaboration network analysis approach for the first time to explore this field.

Through the 3WH framework and the PRISMA method, the databases from the literature were systematically organized and analyzed, from origins to the present, clearly labeling each data in terms of disciplines, methodologies, and species.

Then the analyses quantified multiple aspects of the contributions and evolutions, and the results indicate that the research on the cerebellar role in emotions is at an important transition point. The sustained growth of scientific output and specialization in this area has created significant opportunities while also posing challenges such as knowledge fragmentation. This analysis emphasizes the importance of strengthening ties between disciplines and scientific communities to address these challenges and promote meaningful advancements.

Moreover, the clinical implications associated with the cerebellum's role in emotional regulation highlight new opportunities for understanding and treating neuropsychiatric and emotional disorders. The findings suggest that collaborative and multidisciplinary approaches will be crucial for advancing the understanding of these connections and their practical applications.

The analysis provides a strategic tool for researchers, clinicians, and academics interested in directing their efforts toward high-impact areas. By mapping the connections and networks of scientific collaboration, this study not only facilitates the identification of new opportunities for interdisciplinary partnerships but also fosters the development of innovative projects and optimizes knowledge dissemination. It allows us to understand the origins and evolution of the topic, as well as the factors that influence it.

## Supplementary Information

Below is the link to the electronic supplementary material.

Supplementary file1 [BIB 49890 KB]

Supplementary file2 (RIS 51166 KB)

Supplementary file3 (DOCX 311 KB)

## Data Availability

No datasets were generated or analysed during the current study.

## References

[1] Manto M, Bower JM, Conforto AB, Delgado-García JM, da Guarda SNF, Gerwig M, et al. Consensus paper: roles of the cerebellum in motor control—the diversity of ideas on cerebellar involvement in movement. Cerebellum Lond Engl. 2012;11(2):457–87. 10.1007/s12311-011-0331-9 PMC4347949 22161499

[2] Adamaszek M, D’Agata F, Ferrucci R, Habas C, Keulen S, Kirkby KC, et al. Consensus paper: cerebellum and emotion. Cerebellum. 2017;16(2):552–76. 27485952 10.1007/s12311-016-0815-8

[3] Ciapponi C, Li Y, Osorio Becerra DA, Rodarie D, Casellato C, Mapelli L, et al. Variations on the theme: focus on cerebellum and emotional processing. Front Syst Neurosci. 2023;17:1185752. 37234065 10.3389/fnsys.2023.1185752 PMC10206087

[4] Jacobi H, Faber J, Timmann D, Klockgether T, Jacobi H, Faber J, et al. Update cerebellum and cognition. J Neurol. 2021;268(10):3921–5. 33656586 10.1007/s00415-021-10486-w PMC8463403

[5] Sacchetti B, Scelfo B, Strata P, Sacchetti B, Scelfo B, Strata P. Cerebellum and emotional behavior. Neuroscience. 2009;162(3):756–62. 19409218 10.1016/j.neuroscience.2009.01.064

[6] Van Overwalle F, Manto M, Cattaneo Z, Clausi S, Ferrari C, Gabrieli J, et al. Consensus paper: cerebellum and social cognition. Cerebellum. 2020;19(6):833–68. 32632709 10.1007/s12311-020-01155-1 PMC7588399

[7] Gil-Paterna P, Furmark T. Imaging the cerebellum in post-traumatic stress and anxiety disorders: a mini-review. Front Syst Neurosci. 2023;17. 10.3389/fnsys.2023.1197350/full. 10.3389/fnsys.2023.1197350 PMC10460913 37645454

[8] Chin P, Augustine G. The cerebellum and anxiety. Front Cell Neurosci. 2023;17. 10.3389/fncel.2023.1130505. 10.3389/fncel.2023.1130505 PMC9992220 36909285

[9] Shakiba A. The role of the cerebellum in neurobiology of psychiatric disorders. Neurol Clin. 2014;32(4):1105–15. 25439296 10.1016/j.ncl.2014.07.008

[10] Phillips J, Hewedi D, Eissa A, Moustafa A, Phillips JR, Hewedi DH, et al. The cerebellum and psychiatric disorders. Front PUBLIC Health. 2015. 10.3389/fpubh.2015.00066. 26000269 10.3389/fpubh.2015.00066 PMC4419550

[11] Olivito G, Siciliano L, Clausi S, Lupo M, Baiocco R, Gragnani A, et al. The cerebellum gets social: evidence from an exploratory study of cerebellar, neurodevelopmental, and psychiatric disorders. Biomedicines. 2023;11(2):309. 36830846 10.3390/biomedicines11020309 PMC9953169

[12] Öztürk O, Kocaman R, Kanbach DK. How to design bibliometric research: an overview and a framework proposal. Rev Manag Sci. 2024;18(11):3333–61.

[13] Ajiferuke I, Grácio MCC, Yang S. Editorial: research collaboration and networks: characteristics, evolution and trends. Front Res Metr Anal. 2021;6. 10.3389/frma.2021.690986/full. 10.3389/frma.2021.690986 PMC8137970 34027300

[14] Harris JL, Booth A, Cargo M, Hannes K, Harden A, Flemming K, et al. Cochrane qualitative and implementation methods group guidance series—papers 1–6. J Clin Epidemiol. 2018;1(97):39–48. 10.1016/j.jclinepi.2017.10.023 29248725

[15] PubMed. PubMed. National library of medicine. (n.d.) Available from: https://pubmed.ncbi.nlm.nih.gov/. Accessed 18 Oct 2024.

[16] ScienceDirect. ScienceDirect: science, health, and medical journals, full text articles and books. (n.d.). Available from: https://www.sciencedirect.com/. Accessed 18 Oct 2024.

[17] Scopus. Scopus preview - scopus - welcome to scopus. (n.d.). Available from: https://www.scopus.com/home.uri. Accessed 18 Oct 2024.

[18] Clarivate. Web of Science platform. (n.d.). Available from: https://clarivate.com/products/scientific-and-academicresearch/research-discovery-and-workflow-solutions/webofscience-platform/. Accessed 18 Oct 2024.

[19] Rayyan. Rayyan – intelligent systematic review. (2021). Available from: https://www.rayyan.ai/. Accessed 18 Oct 2024.

[20] Zotero. Zotero: your personal research assistant. (n.d.). Available from: https://www.zotero.org/. Accessed 18 Oct 2024.

[21] Chawla ADS. Retraction watch. 2024. Available from: https://retractionwatch.com/. Accessed 18 Oct 2024.

[22] Schnetter E. eschnett/zotero-citationcounts. 2024. Available from: https://github.com/eschnett/zotero-citationcounts. Accessed 19 Oct 2024.

[23] Clarivate. Journal citation reports (JCR). (n.d.). Available from: https://jcr.clarivate.com. Accessed 20 Oct 2024.

[24] Strotmann A, Zhao D, Bubela T. Author name disambiguation for collaboration network analysis and visualization. Proc Am Soc Inf Sci Technol. 2009;46(1):1–20.

[25] pandas - Python Data Analysis Library. (n.d.). Available from: https://pandas.pydata.org/. Accessed 19 Oct 2024.

[26] Haddow G. Bibliometric research. In: Williamson K, Johanson G, editors. Research methods: information, systems and contexts. Prahran, Vic: Tilde University Press; 2013. p. 219–244. Available from: http://hdl.handle.net/20.500.11937/42722.

[27] Matplotlib. Visualization with python. (n.d.). Available from: https://matplotlib.org/. Accessed 19 Oct 2024.

[28] Waskom M. seaborn: statistical data visualization. J Open Source Softw. 2021;6(60):3021.

[29] Schmahmann JD, Sherman JC. Cerebellar cognitive affective syndrome. In: Schmahmann JD, editor. International review of neurobiology. Academic Press; 1997;(41):433–40. Available from: https://www.sciencedirect.com/science/article/pii/S0074774208603633. Accessed 24 Jan 2025. 10.1016/s0074-7742(08)60363-3 9378601

[30] SpringerLink. The cerebellum. (n.d.). Available from: https://link.springer.com/journal/12311. Accessed 20 Oct 2024.

[31] Elsevier. JAD | Journal of affective disorders. (n.d.). Available from: https://www.sciencedirect.com/journal/journal-of-affective-disorders. Accessed 20 Oct 2024.

[32] VOSviewer. VOSviewer - Visualizing scientific landscapes. (n.d.). Available from: https://www.vosviewer.com/. Accessed 23 Jan 2025.

[33] Massachusetts General Hospital. Jeremy Schmahmann, MD - Department of neurology. (n.d.). Available from: https://www.massgeneral.org/doctors/16489/jeremy-schmahmann. Accessed 21 Oct 2024.

[34] GeoPandas. GeoPandas 1.0.1 — GeoPandas 1.0.1+0.g747d66e.dirty documentation. (n.d.). Available from: https://geopandas.org/en/stable/. Accessed 27 Nov 2024.

[35] NetworkX. NetworkX documentation. (n.d.). Available from: https://networkx.org/. Accessed 30 Oct 2024.

[36] graph-tool. Efficient network analysis with Python. (n.d.). Available from: https://graph-tool.skewed.de/. Accessed 30 Oct 2024.

[37] Barabási AL. Network science. (n.d.). Available from: http://networksciencebook.com/. Accessed 4 Nov 2024.

[38] Arif T. The mathematics of social network analysis: metrics for academic social networks. Int J Comput Appl Technol Res. 2015.

[39] Newman M. Networks. Oxford University Press; 2018. Available from: 10.1093/oso/9780198805090.001.0001. Accessed 11 Apr 2024.

[40] Diestel R. Graph theory. 6th ed. Springer; 2024. Available from: https://diestel-graph-theory.com/. Accessed 2 Dec 2024.

[41] Fu L, Aximu R, Zhao G, Chen Y, Sun Z, Xue H, et al. Mapping the landscape: a bibliometric analysis of resting-state fMRI research on schizophrenia over the past 25 years. Schizophrenia. 2024;10(1):1–8. 38490990 10.1038/s41537-024-00456-2 PMC10942978

[42] Lv M, Feng Y, Zeng S, Zhang Y, Shen W, Guan W, et al. A bibliometrics analysis based on the application of artificial intelligence in the field of radiotherapy from 2003 to 2023. Radiat Oncol. 2024;19(1):1–13. 39529129 10.1186/s13014-024-02551-1 PMC11552138

[43] Jingili N, Oyelere SS, Ojwang F, Agbo FJ, Nyström MBT. Virtual reality for addressing depression and anxiety: a bibliometric analysis. Int J Environ Res Public Health. 2023;20(9):5621. 37174141 10.3390/ijerph20095621 PMC10178384

[44] Wang R, Huang S, Wang P, Shi X, Li S, Ye Y, et al. Bibliometric analysis of the application of deep learning in cancer from 2015 to 2023. Cancer Imaging. 2024;24(1):85. 38965599 10.1186/s40644-024-00737-0 PMC11223420

[45] Emotional Intelligence and Social Interaction – A Bibliometric Analysis. International research journal of multidisciplinary scope (IRJMS). 2024. Available from: https://www.irjms.com/journal/emotional-intelligence-and-social-interaction-a-bibliometric-analysis/. Accessed 23 Jan 2025.

[46] Kuhn TS. The structure of scientific revolutions. Vol. 962. University of Chicago press Chicago; 1997.

[47] Lakatos I. The methodology of scientific research programmes. 1978.

[48] Quine WVO. Two dogmas of empiricism. In: Harding SG, editor. Can theories be refuted? Essays on the Duhem-Quine Thesis. Dordrecht: Springer Netherlands; 1976. p. 41–64. Available from: 10.1007/978-94-010-1863-0_2. Accessed 23 Jan 2025.

[49] D’Angelo E, Jirsa V. The quest for multiscale brain modeling. Trends Neurosci. 2022;45(10):777–90. 35906100 10.1016/j.tins.2022.06.007

[50] Ramaswamy S. Data-driven multiscale computational models of cortical and subcortical regions. Curr Opin Neurobiol. 2024;1(85):102842. 10.1016/j.conb.2024.102842 38320453

[51] Lytton WW, Arle J, Bobashev G, Ji S, Klassen TL, Marmarelis VZ, et al. Multiscale modeling in the clinic: diseases of the brain and nervous system. Brain Inform. 2017;4(4):219–30. 28488252 10.1007/s40708-017-0067-5 PMC5709279

