# Supplementary material for: The Cerebellar Role in Emotions at a Turning Point: Bibliometric Analysis and Collaboration Networks
Source: Cerebellum. 2025 Feb 17;24(2):47. doi: 10.1007/s12311-025-01800-7 (PMC11832776; doi:10.1007/s12311-025-01800-7)
Supplement: Supplementary file 3 — Supplementary file3 (DOCX 311 KB) [file 12311_2025_1800_MOESM3_ESM.docx]

The cerebellar role in emotions at a turning point: Bibliometric analysis and collaboration networks

Dianela A. Osorio-Becerra^1^, Egidio D’Angelo^1,2^ , Claudia Casellato^1,2^

^1^ Department of Brain and Behavioral Sciences, University of Pavia, Pavia, Italy

^2^ Digital Neuroscience Center, IRCCS Mondino Foundation, Pavia, Italy

Supplementary materials

**Table S1: Type of publications***

| **Biology and Medicine Journals 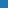**  Acta Medica Portuguesa  Advances in Experimental Medicine and Biology  African Journal of Medicine and Medical Sciences  Aging-Us  American Journal of Medical Genetics  Anales de Pediatría  Archives de Pediatrie  Archives Italiennes de Biologie  Archives of Gerontology and Geriatrics  Archives of Physical Medicine and Rehabilitation  Archivos Argentinos de Pediatría  Biology Letters  Biomed Research International  Biomedical Sciences  Biomedicines  Biorxiv  Brain, Behavior and Evolution  Brain, Behavior, and Immunity  Brazilian Journal of Medical and Biological Research  British Journal of Anaesthesia  British Journal of Hospital Medicine  Bulletin of Experimental Biology and Medicine  Cancer  Case Reports in Medicine  Cell Reports  Child Abuse & Neglect  Clinical Case Reports  Clinical Neurophysiology  Cureus  Current Biology  Developmental Disabilities Research Reviews  Elife  European Journal of Pediatrics  Folia Morphologica  Frontiers in Cell and Developmental Biology  Frontiers in Neurology  Frontiers in Public Health  Hormones and Behavior  Human Molecular Genetics  Internal Medicine  International Journal of Legal Medicine  International Journal of Molecular Sciences  International Journal of MS Care  International Review of Neurobiology  Journal of Cancer Survivorship  Journal of Clinical and Diagnostic Research  Journal of Medical Case Reports  Journal of Neurochemistry  Journal of Pediatric Hematology/ Oncology  Journal of Pediatric Hematology/Oncology Nursing  Journal of Pediatric Rehabilitation Medicine  Journal of the American Geriatrics Society  Journal of Trace Elements in Medicine and Biology  JPMA The Journal of the Pakistan Medical Association  Kaohsiung Journal of Medical Sciences  Medical Hypotheses  Medical Science Monitor  Medicine  Medrxiv  Metabolic Brain Disease  Metabolites  Molecular Neurobiology  Molecular Nutrition & Food Research  Molecular Therapy Nucleic Acids  Nan Fang Yi Ke Da Xue Xue Bao  Nature Communications  Neurobiology of Learning and Memory  Neurobiology of Stress  Neurogenetics  Neuro-Oncology Advances  Neuropsychobiology  Oncotarget  Orvosi Hetilap  Pediatria de Atencion Primaria  Pediatric Hematology and Oncology  Physiological Genomics  Physiological Research  Physiology & Behavior  Plastic and Reconstructive Surgery  Plos One  Proceedings of the National Academy of Sciences of the United States of America  Psychosomatic Medicine  Psychosomatics  Reproductive Sciences  Science  Scientific Reports  Trials  Zhejiang Da Xue Xue Bao Yi Xue Ban  Zhongguo Dang Dai Er Ke Za Zhi  Zhongguo Ying Yong Sheng Li Xue Za Zhi  Zhonghua Yi Xue Za Zhi  **Neuroscience Journals 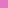**  Aims Neuroscience  American Journal of Play  Annual Review of Neuroscience  Behavioral and Brain Functions  Behavioral Neuroscience  Behavioural Brain Research  Brain Circulation  Brain Connectivity  Brain Research  Brain Research Bulletin  Brain Research Reviews  Brain Sciences  CNS Neuroscience & Therapeutics  CNS Spectrums  Cognitive Affective & Behavioral Neuroscience  Cognitive Neurodynamics  Cognitive, Affective & Behavioral Neuroscience  Developmental Cognitive Neuroscience  eNeuro  Experimental Brain Research  Frontiers in Aging Neuroscience  Frontiers in Behavioral Neuroscience  Frontiers in Cellular Neuroscience  Frontiers in Human Neuroscience  Frontiers in Neural Circuits  Frontiers in Neuroendocrinology  Frontiers in Neuroscience  Frontiers in Synaptic Neuroscience  Frontiers in Systems Neuroscience  IBRO Reports  International Journal of Neuroscience  Journal of Clinical Neuroscience  Journal of Integrative Neuroscience  Journal of Neurolinguistics  Journal of Neuroscience  Journal of Neuroscience Methods  Journal of Neuroscience Research  Nature Neuroscience  Nature Reviews Neuroscience  Neural Plasticity  Neural Regeneration Research  Neurocase  Neuron  Neuroreport  Neuroscience and Biobehavioral Reviews  Neuroscience Letters  Neuroscience Research  Neuroscience  Nutritional Neuroscience  Reviews in the Neurosciences  Social Cognitive and Affective Neuroscience  Social Neuroscience  Synapse (New York, N.Y.)  The Neuroscientist  The European Journal of Neuroscience  The Journal of Neuroscience  Zhurnal Vysshei Nervnoi Deiatelnosti Imeni IP Pavlova  **Pharmacology and Therapies Journals 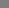**  Archives of Physical Medicine and Rehabilitation  Behaviour Research and Therapy  Biochemical Pharmacology  Brain Stimulation  Cellular Physiology and Biochemistry  Biochemistry, and Pharmacology  Chemosphere  Clinical Psychopharmacology and Neuroscience  Cognitive Therapy and Research  Complementary Therapies in Medicine  Current Neuropharmacology  Environmental Toxicology and Pharmacology  European Journal of Pharmacology  International Journal of Immunopathology and Pharmacology  International Journal of Neuropsychopharmacology  Journal of Child and Adolescent Psychopharmacology  Journal of Clinical Psychopharmacology  Journal of Psychopharmacology  Molecular Therapy Nucleic Acids  Neuropharmacology  Nihon Shinkei Seishin Yakurigaku Zasshi  Physical Medicine and Rehabilitation  Progress in Neuropsychopharmacology & Biological Psychiatry  Psychopharmacology  **Psychology Journals 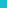**  Acta Psychologica Sinica  Adicciones  Alcohol and Alcoholism  Annales Médico-Psychologiques, Revue Psychiatrique  Applied Neuropsychology: Adult  Applied Neuropsychology: Child  Archives of Clinical Neuropsychology  Australian Journal of Psychology  Child Neuropsychology  Cogent Psychology  Cognition & Emotion  Cognizance of Schizophrenia: A Profound Insight into the Psyche  Current Addiction Reports  Development and Psychopathology  Developmental Neuropsychology  Emotion  European Journal of Psychotraumatology  European Journal of Trauma & Dissociation  Frontiers in Psychology  Indian Journal of Psychological Medicine  International Journal of Clinical and Health Psychology  International Journal of Psychoanalysis  International Journal of Psychology  International Journal of Psychophysiology  Italian Journal of Psychopathology  Journal of Addictive Diseases  Journal of Child Psychology and Psychiatry and Allied Disciplines  Journal of Physiology  Journal of Psychophysiology  Journal of the International Neuropsychological Society  Motivation Science  Neuropsychologia  Neuropsychology Review  Personality and Individual Differences  PhD Existence 2014  Psychological Bulletin  Psychological Medicine  Psychoneuroendocrinology  Psychopathology  Psychophysiology  Psychotherapie Psychosomatik Medizinische Psychologie  Sante Mentale au Quebec  The Clinical Neuropsychologist | **Books** 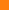  Anatomy of Neuropsychiatry  Anticipation and Medicine  Development of the Cerebellum from Molecular Aspects to Diseases  Developmental Disorders of the Brain  Disorders of Emotion in Neurologic Disease  Essentials of Cerebellum and Cerebellar Disorders  Emotions and the Psychodynamics of the cerebellum  Handbook of Clinical Neurology  Progress in Brain Research \| Book series  Neural Mechanisms of Language  Pattern Analysis of the Human Connectome  The Emotional Cerebellum  The Cerebellum Disorders and Treatment  The Linguistic Cerebellum  Understanding Emotions  **Congresses, Conferences and**  **Meetings 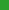**  18th European Congress of Psychiatry  2013 International Conference on Advanced Computer Science Applications and Technologies  17th International Computer Conference on Wavelet Active Media Technology and Information Processing  Abstract of the 25th European Congress of Psychiatry  Abstract Supplement  Abstracts of the 14 European Congress of Clinical Neurophysiology and the 4 International Conference on Transcranial Magnetic and Direct Current Stimulation Neuroimage: Clinical  Abstracts of the 20th European Congress of Psychiatry  Abstracts of the 21st European Congress of Psychiatry  Abstracts of the 22nd European Congress of Psychiatry  Abstracts of the 24th European Congress of Psychiatry  Interspeech 2009: 10th Annual Conference of The International Speech Communication Association  Journées de Neurologie de Langue Française 2017  Annual International Conference of The IEEE Engineering in Medicine and Biology Society  Proceedings of the 10th International Child Neurology Congress  Evolution and Development of Nervous System  The Abstracts from the 2024 Southern Regional Meeting  IBRO World Congress  **Medical Technology and Neurocomputing Journals 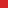**  Acta Polytechnica Hungarica  Adaptive Behavior  Applied Biochemistry and Biotechnology  Applied Sciences-Basel  BMC Medical Imaging  Brain Imaging and Behavior  Brain Stimulation  Chinese Journal of Medical Imaging Technology  Computational and Mathematical Methods in Medicine  European Journal of Radiology  European Radiology Experimental  Hellenic Journal of Nuclear Medicine  Human Brain Mapping  IEEE Transactions on Neural Systems and Rehabilitation Engineering  International Journal of Fuzzy Systems  International Journal of Nanotechnology  Journal of Hazardous Materials  Journal of Neuroimaging  Neurocomputing  Neuroimage Clinical  Neuroimage  Neuroinformatics  Plos Computational Biology  Scientific Programming  Statistics in Medicine  **Neurology Journals 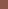**  Acta Neurochirurgica  Acta Neurologica Belgica  Acta Neurológica Latinoamericana  Alzheimer's & Dementia  Annals of Indian Academy of Neurology  Annals of Neurology  Archives of Neurology  Archives of Pediatric Neurosurgery  Archivos Argentinos de Pediatría  Behavioral and Brain Functions  Behavioural Brain Research  Behavioural Neurology  Biological Psychiatry-Cognitive Neuroscience and Neuroimaging  BMC Neurology  Brain & Development  Brain: A Journal of Neurology  Brain and Behavior  Brain and Cognition  Brain and Nerve = Shinkei Kenkyu no Shinpo  Brain Injury  Brain Structure & Function  Brain  British Journal of Neurosurgery  Cerebellum & Ataxias  Cerebellum  Cerebral Cortex  Cerebral Cortex Communications  Cerebrovascular Diseases  Child's Nervous System  Clinical Neurology and Neurosurgery  Cognitive and Behavioral Neurology  Cortex  Current Neurology and Neuroscience Reports  Current Opinion in Behavioral Sciences  Current Opinion in Neurology  Dementia  Der Nervenarzt  Developmental Medicine and Child Neurology  Epilepsia  Epilepsy & Behavior Reports  Epilepsy & Behavior  European Journal of Neurology  European Journal of Paediatric Neurology  Frontiers in Neurology  Frontiers in Physiology  Genes, Brain, and Behavior  Journal of Alzheimer's Disease  Journal of Clinical Neurology (Seoul Korea)  Journal of Geriatric Psychiatry and Neurology  Journal of Intellectual Disability Research  Journal of Neural Transmission  Journal of Neurological Surgery Part B: Skull Base  Journal of Neurology Neurosurgery and Psychiatry  Journal of Neurology  Journal of Neurology, Neurosurgery, and Psychiatry  Journal of Neurosurgery  Journal of Neurosurgery: Pediatrics  Journal of Stroke & Cerebrovascular Diseases  Journal of The Neurological Sciences  L.O.G.O.S. Interdisziplinair  Movement Disorders  Multiple Sclerosis  Nature Reviews Neuroscience  Nervenarzt  Neurologia I Neurochirurgia Polska  Neurologic Clinics  Neurological Research and Practice  Neurological Research  Neurological Sciences  Neurology Asia  Neurology India  Neurology Perspectives  Neurology  Neurooncology Advances  Neurooncology  Parkinsonism & Related Disorders  Pediatric Neurology  Pediatric Neurosurgery  Perceptual and Motor Skills  Progress in Neurological Surgery  Revista de Neurologia  Revue Neurologique  Surgical Neurology International  The Primary Care Companion for CNS Disorders  The Cerebellum  World Neurosurgery  Zhurnal Nevrologii I Psikhiatrii Imeni S.S. Korsakova  **Psychiatry Journals 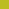**  Acta Neuropsychiatrica  Acta Psychiatrica Scandinavica  Actas Españolas De Psiquiatría  American Journal of Geriatric Psychiatry  American Journal of Psychiatry  Archives of General Psychiatry  Arquivos de Neuropsiquiatria  Asia-Pacific Psychiatry  Australian and New Zealand Journal of Psychiatry  Biological Psychiatry: Cognitive Neuroscience and Neuroimaging  Biological Psychiatry Global Open Science  Biological Psychiatry  Bipolar Disorders  BMC Psychiatry  British Journal of Psychiatry  Canadian Journal of Psychiatry-Revue Canadienne De Psychiatrie  Case Reports in Psychiatry  Ceskoslovenska Psychiatrie  Chronic Stress  Clinical Schizophrenia and Related Psychoses,  Cognizance of Schizophrenia: A Profound Insight into the Psyche  Current Psychiatry Reports  Current Psychiatry Research and Reviews  Dementia & Neuropsychologia  Depression and Anxiety  Epidemiologia e Psichiatria Sociale  European Archives of Psychiatry and Clinical Neuroscience  European Psychiatry  Frontiers In Psychiatry  General Psychiatry  International Journal of Psychiatry in Clinical Practice  International Review of Psychiatry  JAMA Psychiatry  Journal of Affective Disorders  Journal of Clinical Psychiatry  Journal of Nervous and Mental Disease  Journal of Neurodevelopmental Disorders  Journal of Neuropsychiatry and Clinical Neurosciences  Journal of Psychiatric Research  Journal of Psychiatry & Neuroscience  Journal of the American Academy of Child and Adolescent Psychiatry  Molecular Psychiatry  Neurochemical Research  Neurochemistry International  Neuropsychiatric Disease and Treatment  Noropsikiyatri Arsivi  Praxis  Psiquiatría Danubina  Psiquiatría Polska  Psychiatric Annals  Psychiatry And Clinical Neurosciences  Psychiatry Investigation  Psychiatry Research  Psychiatry Research: Neuroimaging  Revista Brasileira de Psiquiatria  Revista Colombiana de Psiquiatría  Revista de Psiquiatria Clinica  Rivista di Psichiatria  Schizophrenia  Schizophrenia Bulletin  Schizophrenia Research  Suicide & Life-Threatening Behavior  The American Journal of Geriatric Psychiatry  The Canadian Journal of Psychiatry  The British Journal of Psychiatry  The Journal of Neuropsychiatry and Clinical Neurosciences  Tijdschrift Voor Psychiatrie  Translational Psychiatry  World Journal of Biological Psychiatry |
| --- | --- |
|  |  |

* Owing to the complex nature of the brain and human behavior, it is common for journals in this field of study to cover a wide range of fields, as boundaries between fields are often fluid and research can be interdisciplinary. In other words, a journal may publish articles related to neuroscience, psychiatry, and medical technologies at the same time; in such cases, we opt for the thematic approach as a measure to classify them.

**Table S2: Leading Journals**

| **Leading Journals** | | |
| --- | --- | --- |
| **Publication Title** | **JIF** | **Quartile** |
| Cerebellum | 2.7 | Q3 |
| Journal of Affective Disorders | 4.9 | Q1 |
| Plos One | 2.9 | Q1 |
| Psychiatry Research-Neuroimaging | 2.1 | Q3 |
| Neuroimage | 4.7 | Q1 |
| Human Brain Mapping | 3.5 | Q1 |
| Psychological Medicine | 5.9 | Q1 |
| Frontiers in Psychiatry | 3.2 | Q2 |
| Social Cognitive and Affective Neuroscience | 3.9 | Q1 |
| Biological Psychiatry | 9.6 | Q1 |
| Neuroimage-Clinical | 3.4 | Q2 |
| BRAIN | 10.6 | Q1 |
| Scientific Reports | 3.8 | Q1 |
| Brain Imaging and Behavior | 2.4 | Q2 |
| Neuropsychologia | 2.0 | Q3 |
| Cortex | 3.2 | Q1 |
| Neuroscience Letters | 2.5 | Q3 |
| Neuroreport | 1.6 | Q4 |
| Progress in Neuro-Psychopharmacology & Biological Psychiatry | 5.3 | Q1 |
| Translational Psychiatry | 5.8 | Q1 |
| Behavioural Brain Research | 2.6 | Q2 |
| Neuroscience | 2.9 | Q2 |
| Journal of Psychiatric Research | 3.7 | Q1 |
| Journal of Neuropsychiatry and Clinical Neurosciences | 2.4 | Q2 |
| Movement Disorders | 7.4 | Q1 |
| Journal of Psychiatry & Neuroscience | 4.1 | Q1 |
| Frontiers In Behavioral Neuroscience | 2.6 | Q2 |
| Molecular Psychiatry | 9.6 | Q1 |
| Neurology | 7.7 | Q1 |

Citation, impact factor (IF) and quartile (Q) are widely used metrics in bibliometric analysis. The impact factor measures the average number of citations that a journal's articles receive in a given period, while the quartile positions the journal in a relative ranking within its area of expertise. Although these metrics are useful for evaluating the relevance, visibility, and influence of research and publications, they do not capture all aspects of scientific quality. Therefore, they are presented as complementary information for broader analysis.

A notable aspect of the table is that most of the leading journals are located in the first quartile (Q1), which indicates that they are above 75% of the journals in their category. This quartile groups the journals with the highest impact factor (JIF) in that category.

**Table S3: Leading cited publications**

| **Title** | **Journal** | **Authors** |
| --- | --- | --- |
| Empathy for pain involves the affective but not sensory components of pain | Science  Vol 303, Issue 5661 pp. 1157-1162 | T Singer, B Seymour, J O'Doherty, H Kaube, RJ Dolan, and CD Frith |
| Cerebellar cognitive affective syndrome | Brain  Apr:121 ( Pt 4):561-79 | JD Schmahmann and JC Sherman |
| Cerebellum and Non-motor Function | Annual review of neuroscience  Vol. 32:413-434 | PL Strick, RP Dum, and JA Fiez |
| Disorders of the cerebellum: Ataxia, dysmetria of thought, and the cerebellar cognitive affective syndrome | The Journal of Neuropsychiatry and Clinical Neurosciences  Volume 16, Number 3 | JD Schmahmann |
| The functional neuroanatomy of autobiographical memory: A meta-analysis | Neuropsychologia  44(12):2189-208 | E Svoboda, MC McKinnon, and B Levine |
| Evidence for topographic organization in the cerebellum of motor control versus cognitive and affective processing | Cortex  46(7):831-44 | CJ Stoodley, and JD Schmahmann |
| Neuroanatomical correlates of pleasant and unpleasant emotion | Neuropsychologia  35(11):1437-44 | RD Lane, EM Reiman, MM Bradley, PJ Lang, GL Ahern, RJ Davidson, and GE Schwartz |
| A meta-analytic study of changes in brain activation in depression | Human Brain Mapping  29(6):683-95 | PB Fitzgerald, AR Laird, J Maller, and ZJ Daskalakis |
| The anatomy of mood disorders - Review of structural neuroimaging studies | Biological Psychiatry  1;41(1):86-106 | JC Soares, and JJ Mann |
| The functional neuroanatomy of bipolar disorder: a review of neuroimaging findings | Molecular Psychiatry  10(1):105-16 | SM Strakowski, MP Delbello, and CM Adler |
| Attenuation of the neural response to sad faces in major depression by antidepressant treatment - A prospective, event-related fMRI study | Archives of General Psychiatry  61(9):877-89 | CHY Fu, SCR Williams, AJ Cleare, MJ Brammer, ND Walsh, J Kim, CM Andrew, EM Pich, PM Williams, LJ Reed, MT Mitterschiffthaler, J Suckling, and ET Bullmore |
| Dissociation of Neural Representation of Intensity and Affective Valuation in Human Gustation | Neuron  14;39(4):701-11 | DM Small, MD Gregory, YE Mak, D Gitelman, MM Mesulam, and T Parrish |
| Neuropsychological consequences of cerebellar tumour resection in children - Cerebellar cognitive affective syndrome in a paediatric | Brain  123(5), 1041-1050 | L Levisohn, A Cronin-Golomb, and JD Schmahmann |
| Identifying major depression using whole-brain functional connectivity: a multivariate pattern analysis | Brain  135(Pt 5):1498-507 | LL Zeng, H Shen, L Liu, L Wang, B Li, P Fang, Z Zhou, Y Li, and D Hu |
| The neuropsychiatry of the cerebellum - insights from the clinic | Cerebellum  6(3):254-67 | JD Schmahmann, JB Weilburg and JC Sherman |
| The Role of the Cerebellum in Cognition and Emotion: Personal Reflections Since 1982 on the dysmetria of thought hypothesis, and its historical evolution from theory to therapy | Neuropsychology Review  20(3):236-60 | JD Schmahmann |
| Empathy and judging other's pain: An fMRI study of alexithymia | Cerebral Cortex  17(9):2223-34 | Y Moriguchi, J Decety, T Ohnishi, M Maeda, T Mori, K Nemoto, HMatsuda, and G Komaki |
| Common and distinct patterns of grey-matter volume alteration in major depression and bipolar disorder: evidence | Molecular Psychiatry 22(10):1455-1463 | T Wise, J Radua, E Via, N Cardoner, O Abe, TM Adams, F Amico, Y Cheng, JH Cole, C de Azevedo Marques Périco, DP Dickstein, TFD Farrow, T Frodl, G Wagner, IH Gotlib, O Gruber, BJ Ham, DE Job, MJ Kempton, MJ Kim, PCMP Koolschijn, GS Malhi, D Mataix-Cols, AM McIntosh, AC Nugent, JT O'Brien, S Pezzoli, ML Phillips, PS Sachdev, G Salvadore, S Selvaraj, AC Stanfield, AJ Thomas, MJ van Tol, NJ A van der Wee, DJ Veltman, AH Young, CH Fu, AJ Cleare, and D Arnone |
| Pathological laughter and crying: a link to the cerebellum | Brain  124(Pt 9):1708-19 | J Parvizi, SW Anderson, CO Martin, H Damasio, and AR Damasio |
| Consensus Paper: Cerebellum and Emotion | Cerebellum  16(2):552-576 | M Adamaszek, F D'Agata, R Ferrucci, C Habas, S Keulen, KC Kirkby, M Leggio, P Mariën, M Molinari, E Moulton, L Orsi, F Van Overwalle, C Papadelis, A Priori, B Sacchetti, DJ Schutter, C Styliadis, and J Verhoeven |
| The emotional power of music: How music enhances the feeling of affective pictures | Brain Research 23;1075(1):151-64 | T Baumgartner, K Lutz, CF Schmidt, and L Jäncke |
| Cerebellum development during childhood and adolescence: A longitudinal morphometric MRI study | Neuroimage  1;49(1):63-70 | H Tiemeier, RK Lenroot, DK Greenstein, LTran, R Pierson, and JN Giedd |
| Emotional valence modulates brain functional abnormalities in depression: Evidence from a meta-analysis of fMRI studies | Neuroscience & Biobehavioral Reviews  37(2), 152-163 | NA Groenewold, EM Opmeer, PJA Aleman, and SG Costafreda |
| Identification of emotional intonation evaluated by fMRI | Neuroimage  15;24(4):1233-41 | D Wildgruber, A Riecker, I Hertrich, M Erb, W Grodd, T Ethofer, and H Ackermann |
| Dissociable Neural Pathways Are Involved in the Recognition of Emotion in Static and Dynamic Facial Expressions | Neuroimage  18(1):156-68 | CD Kilts, G Egan, DA Gideon, TD Ely, and JM Hoffman |
| Neuroanatomical correlates of a lactate-induced anxiety attack | Archives of General Psychiatry  46(6):493-500 | EM Reiman, ME Raichle, E Robins, MA Mintun, MJ Fusselman, PT Fox, JL Price, and KA Hackman |
| Cerebral blood flow changes associated with attribution of emotional valence to pleasant, unpleasant, and neutral visual stimuli in a PET study of normal subjects | The American Journal of Psychiatry 156(10):1618-29. | S Paradiso, DL Johnson, NC Andreasen, DS O'Leary, GL Watkins, LL Ponto, and RD Hichwa |
| Disorders of cognitive and affective development in cerebellar malformations | Brain  130(Pt 10):2646-60 | A Tavano, R Grasso, C Gagliardi, F Triulzi, N Bresolin, F Fabbro, and R Borgatti |
| Incidence and severity of postoperative cerebellar mutism syndrome in children with medulloblastoma: a prospective study by the Children's Oncology Group | Journal of Neurosurgery  105(6 Suppl):444-51 | PL Robertson, KM Muraszko, EJ Holmes, R Sposto, RJ Packer, A Gajjar, MS Dias, JC Allen; Children's Oncology Group |

**Fig. S1 Role of the authors**

**
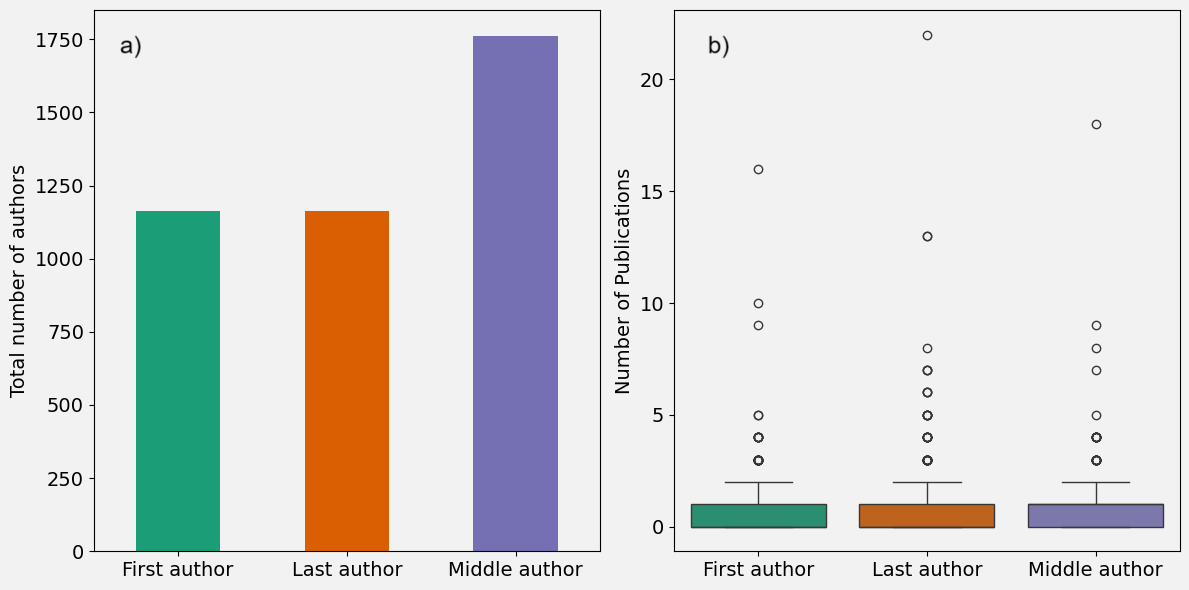
**

a) Displays a bar chart showing the total number of authors by role, with bar length indicating the number of authors, allowing for quick visual comparison. b) Features of a box plot illustrating the distribution of authors by role in each publication. Each box shows the interquartile range, the line inside indicates the median, and the whiskers represent the data range, while points outside the whiskers are outliers.

The first author typically carries out the bulk of the research and writing, signaling their leadership in the study, whereas the last author often represents the principal investigator, responsible not only for supervision but also for the management of the project and its funding. On the other hand, intermediate researchers participate in key aspects such as data collection and analysis, experiments, or providing specific knowledge.

To better understand the roles of authors, we used two complementary graphs. Panel a) shows that the most predominant role is the middle author, suggesting that publications frequently include multiple middle authors, which is typical for collaborative papers involving larger teams. Importantly, an author can be the first author in one publication and the middle or last author in another. Therefore, when counting contributions in each position, more than one can be added for the same author.

On the other hand, on Panel b), we see that for each role, most authors have on average, two publications (the range represented by the box). The circles that are scattered above the whiskers represent authors who have a significantly greater number of publications than the majority.

The circles range from darkest to least dark according to the number of authors in that range. There are a greater number of authors who have published 3 or 4 papers, while there are significantly fewer authors with 5 or more publications, with some authors reaching more than 10 publications and one with more than 20 publications, as in the case of the last author.

To identify authors who belong to these outside-the-mean circles (the outliers, which are shown outside the box) in the previous figure, we used a bubble chart (Fig. S2). This allows for clear visualization of the authors who have a significantly greater number of publications than the majority do.

**Fig. S2 Extended version of Fig. S1, highlighting authors outside the mean**


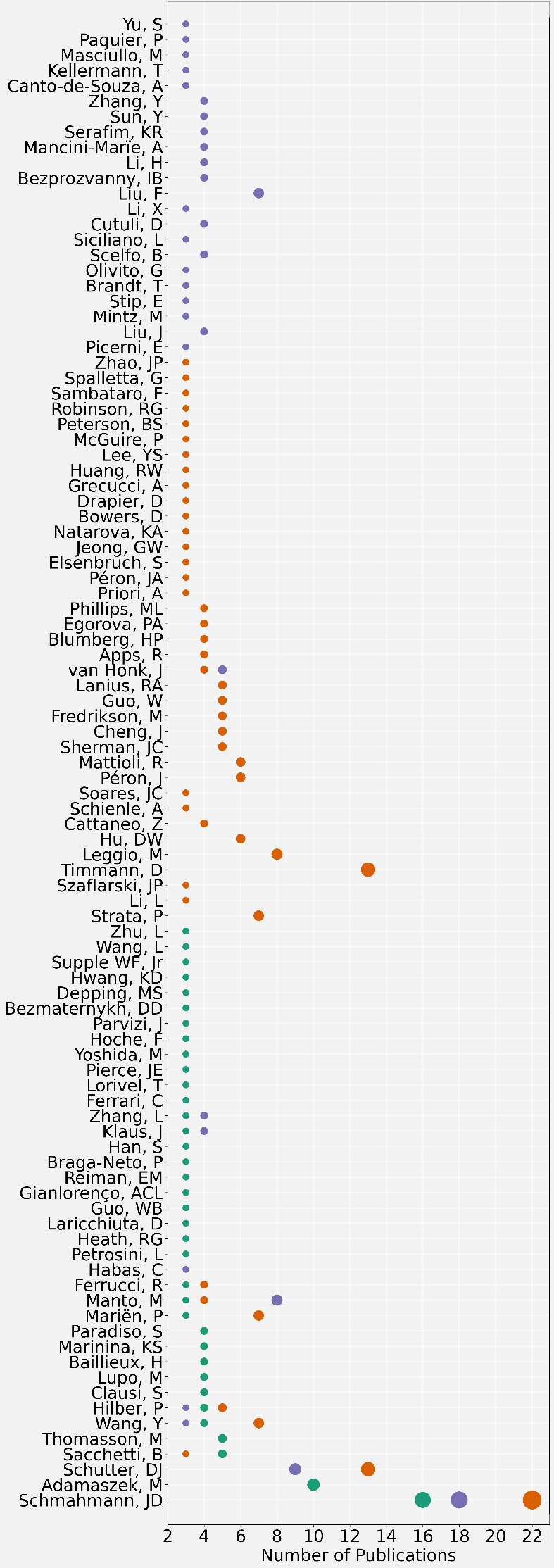


The Y-axis lists the authors' names, while the X-axis indicates the number of publications attributed to each author. Additionally, the size of the bubbles represents the number of publications; larger bubbles correspond to a greater number of publications. The colours of the bubbles match those in the previous figure, distinguishing between the first, last, and middle authors

**Table S4 Network Metrics**: Evolutionary and Comparative Analysis**

| **Period** | **1961-2000** | **1961-2012** | **1961-2018** | **1961-2024** |
| --- | --- | --- | --- | --- |
| **Publications and Collaborations** | | | | |
| Number of publications | 66.0 | 351 | 676 | 1.16E3 |
| Total Collaborations | 1.51E3 | 1.06E4 | 2.61E4 | 6.33E4 |
| Average number of collaborations per author (Average degree of connectivity) | 5.56 | 6.78 | 8.28 | 10.9 |
| **Network and Connectivity** | | | | |
| Number of nodes | 271 | 1.57E3 | 3.15E3 | 5.82E3 |
| Number of links | 772 | 5.77E3 | 1.43E4 | 3.39E4 |
| Connected graph | No | No | No | No |
| Most connected node | Reiman, EM | Wiesmann, M | Phillips, ML | Phillips, ML |
| Giant Cluster | 17.0 | 52.0 | 536 | 2.71E3 |
| **Efficiency and Paths** | | | | |
| Characteristic Path Length | 1.92 | 2.34 | 4.84 | 7.80 |
| Global Efficiency | 2.31E-2 | 6.06E-3 | 1.32E-2 | 3.50E-2 |
| Average Distance | 1.92 | 2.34 | 4.84 | 7.80 |
| Network Diameter | 4.00 | 3.00 | 12.0 | 18.0 |
| Average Length of Shortest Path | 1.92 | 2.34 | 4.84 | 7.80 |
| **Centrality** | | | | |
| Average Degree Centrality | 2.06E-2 | 4.33E-3 | 2.63E-3 | 1.87E-3 |
| Average Betweenness Centrality | 2.56E-5 | 3.84E-6 | 5.06E-5 | 2.53E-4 |
| Average Closeness Centrality | 2.20E-2 | 5.55E-3 | 1.12E-2 | 2.91E-2 |
| Average Eigenvector Centrality | 1.28E-2 | 8.74E-2 | 2.48E-3 | 1.81E-3 |
| **Clusters and Communities** | | | | |
| Total Communities | 43.0 | 203 | 328 | 502 |
| Average Community Size | 6.30 | 7.73 | 9.60 | 11.6 |
| Total Cliques | 56.0 | 314 | 618 | 1.10E3 |
| Average Cliques Size | 5.18 | 5.87 | 6.24 | 6.74 |
| Average Clustering Coefficient | 0.92 | 0.93 | 0.92 | 0.92 |
| **Diversity and Modulation** | | | | |
| Diversity and Degree Entropy | 3.25 | 3.76 | 4.09 | 4.32 |
| Modularity | 0.96 | 0.99 | 0.95 | 0.91 |
| **Density and Resilience** | | | | |
| Network Density | 2.06E-2 | 4.33E-3 | 2.63E-3 | 1.87E-3 |
| Assortativity | 0.84 | 0.58 | 0.64 | 0.92 |
| Resilience (degree of nodes) | 17.0 | 52.0 | 536 | 2.71E3 |
| Vulnerability (degree of nodes) | 43.0 | 203 | 318 | 474 |
| Resilience (random) | 0.36 | 0.35 | 0.34 | 0.40 |
| Vulnerability (random) | 0.64 | 0.65 | 0.66 | 0.60 |

**For more details on how each metric is calculated, refer to (1,2)

The metrics quantitively confirm the observations in Fig. 15. The network has experienced significant growth in publications, authors, and collaborations, indicating increased interest and relevance in this field within the scientific community. Publications have risen exponentially from 66.0 to 1.16E3, accompanied by a substantial increase in the number of nodes from 271 to 5.82E3, indicating that more researchers are joining and diversifying the collaborative network. Links between authors have grown even faster, from 772 to 3.39E4, reflecting more frequent and diverse collaborations. The average number of collaborations per author nearly doubled from 5.56--10.9, demonstrating strengthened coauthorship and network cohesion. This complexity suggests interdisciplinary collaboration and enriched interactions among researchers.

The network's most connected nodes change over time, with “Reiman, MS” and “Wiesmann, M” initially leading and “Phillips, ML” becoming the dominant node in later periods, playing a critical role in knowledge dissemination. The giant cluster grows significantly, from 17.0 to 2.71E3 nodes, reflecting increased collaboration and cohesion in the network. Higher average connections and giant cluster expansion create a more robust, stable, and resilient network. The global efficiency improves, reaching 3.50E-2, enhancing internode communication. However, the characteristic path length increases from 1.92 to 7.80, and the network diameter expands from 4.00 to 18.0, indicating greater dispersion and complexity in collaborative paths as the network grows.

The network's growth shows diversification and increasing collaboration. Degree centrality decreases steadily, reflecting a less dense and more fragmented structure, whereas betweenness centrality slightly increases, indicating a greater influence of certain nodes as bridges. Closeness centrality initially decreases but later increases, showing improved cohesion and shorter paths for communication. Eigenvector centrality starts at 1.28E-2, peaks at 8.74E-2, and then decreases to 2.48E-3 and 1.81E-3, indicating a shift from concentrated influence to a more evenly distributed structure. The number of communities grows from 43.0--502, with the average community size increasing from 6.30--11.6. Similarly, the number of cliques increases from 56.0 to 1.10E3, with the average size increasing from 5.18 to 6.74. These trends reflect a network that, despite its expansion, fosters specialized, cohesive, and efficient scientific collaboration.

The degree entropy increases from 3.25 to 4.32, indicating greater diversity in connections, as new authors join or existing ones collaborate with a wider, more interdisciplinary group. Modularity decreases slightly from 0.96 to 0.91, suggesting that the network remains highly modular but becomes more integrated, with diminishing barriers between subgroups.

The network density decreases from 2.06E-2 to 1.87E-3, as not all new authors establish connections, reflecting a sparser structure. Rising assortativity suggests that more connected nodes collaborate, fostering impactful projects but concentrating collaboration among core researchers. The giant component remains central to communication and information flow. Over time, the network shows increased resilience but also greater vulnerability to the removal of key nodes, indicating a growing dependence on influential participants. Random node removal initially reduces resilience and increases vulnerability but improves by 2024, highlighting a trend toward greater robustness to random failures while remaining reliant on critical nodes.

References

1. networkx/networkx [Internet]. NetworkX; 2025 [cited 2025 Jan 24]. Available from: https://github.com/networkx/networkx

2. GitLab [Internet]. 2025 [cited 2025 Jan 24]. Tiago Peixoto / graph-tool · GitLab. Available from: https://git.skewed.de/count0/graph-tool
